# Effects of Hatha Yoga vs Physical Conditioning on Sleep in Women With Urinary Incontinence: A Secondary Analysis of a Randomized Clinical Trial

**DOI:** 10.1001/jamanetworkopen.2025.46499

**Published:** 2025-12-08

**Authors:** Ellie Hough, Lizabeth A. Goldstein, Leslee L. Subak, Margaret A. Chesney, Michael Schembri, Harini Raghunathan, Sarah Balys Pawlowsky, Alison J. Huang

**Affiliations:** 1 School of Medicine, University of California, San Francisco; 2 Department of Psychiatry and Behavioral Sciences, University of California, San Francisco; 3 San Francisco VA Health Care System, San Francisco, California; 4 Stanford University, Palo Alto, California; 5 Department of Medicine, University of California, San Francisco; 6 Department of Physical Therapy, San Francisco State University, San Francisco, California

## Abstract

**Importance:**

Yoga, an integrative health practice combining movement and mindfulness, has been recommended to improve sleep in adults with chronic conditions, but it is unclear whether it offers unique benefits for sleep compared with nonspecific exercise.

**Objective:**

To compare the effects of a yoga intervention vs a nonspecific physical conditioning intervention on sleep outcomes in women with urinary incontinence.

**Design, Setting, and Participants:**

This prespecified secondary analysis of a multicenter, investigator-blinded randomized clinical trial was conducted from 2018 to 2022, with 3 months of intervention follow-up, at 3 sites in Northern California. Interventions were delivered in person and via videoconference after the start of the COVID-19 pandemic. Women aged 45 to 90 years with daily incontinence were included. Data were analyzed from June to September 2024.

**Interventions:**

Random assignment to a 3-month yoga intervention involving twice-weekly instruction and once-weekly self-directed practice of hatha yoga techniques vs a time-equivalent physical conditioning intervention.

**Main Outcomes and Measures:**

Sleep quality and disruption were assessed using the Pittsburgh Sleep Quality Index (PSQI) and Pittsburgh Sleep Diary. Nocturnal incontinence and voiding frequency were evaluated using voiding diaries.

**Results:**

Among 240 participants (mean [SD] age, 62.0 [8.7] years; 40 Asian [21.1%], 19 Black [7.9%], 160 White [66.7%], and 20 >1 race [8.3%]; 32 Latina [13.3%]), 133 participants (55.4%) reported poor sleep quality (PSQI >5) and 133 of 239 participants with data (55.7%) reported awakening at least once per night to use the bathroom at baseline. The mean change in PSQI score over 3 months was 0.37 points (95% CI, 0.78 to −0.04 points) in the yoga group, vs 0.66 points (95% CI, 1.07 to 0.25 points) in the physical conditioning group (between-group difference: 0.29 points [95% CI −0.28 to 0.86 points]). The mean change in wakefulness after sleep onset was 3.82 minutes (95% CI, 8.15 to −0.52 minutes) in the yoga group, and 6.97 minutes (95% CI, 11.20 to 2.74 minutes) in the physical conditioning group (between-group difference, 3.16 minutes [95% CI, −2.84 to 9.16 minutes]). Across all participants regardless of intervention, greater nocturnal voiding frequency was associated with worse sleep quality; for example, the estimated mean PSQI score was 5.83 points (95% CI, 5.35 to 6.32 points) among participants with fewer than 1 nocturnal voiding episode per night but 6.66 points (95% CI, 6.00 to 7.33 points) among those with at least 2 episodes per night (*P* = .02 for linear trend across increasing categories of nocturnal voiding frequency).

**Conclusions and Relevance:**

In this study, a 3-month yoga intervention did not yield superior outcomes for sleep quality or disruption compared with a general physical conditioning intervention among women with incontinence.

**Trial Registration:**

Ancillary Study of the Lessening Incontinence With Low-Impact Activity Study, ClinicalTrials.gov Identifier: NCT04776720; A Group-Based Therapeutic Yoga Intervention for Urinary Incontinence in Ambulatory Older Women, ClinicalTrials.gov Identifier: NCT03672461

## Key Points

**Question:**

Does the practice of yoga offer unique benefits for sleep in women with urinary incontinence?

**Findings:**

In this secondary analysis of a randomized clinical trial including 240 women at midlife and older with urinary incontinence, a 3-month yoga program involving group instruction and self-directed practice of hatha yoga techniques did not improve sleep quality or disruption more than a time-equivalent program of nonspecific physical conditioning exercises.

**Meaning:**

Despite recommendations to practice yoga to improve sleep in adults with chronic conditions, these findings do not indicate that yoga offers a unique benefit for sleep compared with other, nonspecific forms of exercise.

## Teaser

This secondary analysis of a randomized clinical trial investigates the effects of hatha yoga compared with nonspecific physical conditioning on sleep outcomes in women with urinary incontinence.

## Introduction

Sleep plays a critical role in maintaining health, with poor physical and mental health outcomes reported in individuals with poor-quality or disrupted sleep.^1,2,3^ A wide array of aging-related factors, including changes in underlying circadian rhythms, environmental exposures, overlapping health conditions, and use of health interventions, can influence sleep.^4,5,6^ Behavioral factors, such as physical activity, can be potent modulators of sleep, but as people age, their level of physical activity tends to decrease.^7,8^ Specific nocturnal urinary symptoms, such as nocturia or nocturnal incontinence, can also become more common with aging and can disrupt sleep in older adults.^9^

Aging adults also face challenges identifying effective and safe interventions to improve sleep. Although pharmacologic treatments, such as sedative-hypnotic drugs, can improve sleep onset or duration, they are associated with potentially severe adverse effects in older adults, including delirium, oversedation, and increased falls and fractures.^10^ Consequently, there is increasing interest in identifying behavioral or integrative health interventions that are safer but also address more multifactorial factors influencing sleep.^11^

Yoga is a complementary practice that has been recommended for multiple symptoms and conditions associated with aging.^12,13^ As of 2022, 1 in 6 US adults were practicing some form of yoga for health indications, including up to 10% of adults at midlife and older.^14,15^ As an integrative health practice that can blend physical movement and mindfulness through meditation, breath work, and postures (asanas), yoga is hypothesized to reduce psychophysiological arousal and increase mindfulness,^16^ with the potential to improve sleep in individuals with chronic conditions.^17^

However, there is limited evidence of the benefit of yoga for sleep, particularly in comparison with other physical activities. While multiple studies have reported improvement in sleep quality with yoga practice, few have used rigorous study designs, robust control interventions, or multidimensional sleep measures.^18^ Furthermore, past studies have not examined potential mechanisms underlying the hypothesized effects of yoga interventions on sleep.

This study presents sleep outcome data from a multicenter randomized clinical trial of a yoga intervention vs a physical conditioning intervention for women at midlife and older with urinary incontinence. The yoga intervention was hypothesized to offer beneficial effects on sleep through effects on nocturnal incontinence or other mechanisms. In addition to reporting intervention effects on sleep quality, duration, disruption, and efficiency as prespecified secondary and ancillary outcomes, we assessed whether improvements in sleep could be explained by improvements in urinary symptoms.

## Methods

### Study Design

The Lessoning Incontinence Through Low Impact Activity (LILA) study was a multisite, parallel-group randomized clinical trial of a group-based yoga intervention vs nonspecific physical conditioning intervention in middle-aged and older women with urinary incontinence. As described previously,^19^ participants were ambulatory women aged 45 years or older from Northern California who had daily episodes of stress, urgency, or mixed-type incontinence and were willing to avoid using other clinical incontinence treatments during the study. Candidates had to be able to walk 2 blocks on level ground and could not have complicated urologic histories, such as urologic surgery, cancer, or radiation treatment. In addition, women were excluded if they had participated in organized yoga classes or physical conditioning classes within the past 3 months.

Participants provided informed consent at the screening visit according to the trial protocol (Supplement 1) approved by the University of California, San Francisco Institutional Review Board. Upon enrollment, participants were randomly assigned in equal ratios to participate in the yoga or physical conditioning intervention. Randomization was performed by clinical coordinators using a computer-generated, permuted block scheme with blocks of 2 and 4 participants stratified by predominant clinical urinary incontinence type and clinical site. Investigators and study personnel responsible for abstracting or adjudicating outcomes were blinded, although participants and study personnel delivering or monitoring interventions were aware of intervention assignment. This study is reported following the Consolidated Standards of Reporting Trials (CONSORT) reporting guideline.

### Interventions

The group-based yoga intervention focused on alignment-based hatha yoga techniques selected by a panel of yoga consultants for their potential to improve pelvic floor function, general physical function, and psychological and associated autonomic balance in older women who were ambulatory. The yoga program, informed by Iyengar yoga principles, focused on 16 standard poses widely used in yoga practices, although instruction promoted awareness of pelvic floor structures during practice. Instructors had at least 2 years of yoga teaching experience in the community^19^ and received additional training in tailoring yoga postures to improve pelvic floor function. Participants assigned to yoga attended 90-minute group classes twice weekly and were asked to practice outside of class at least once a week during the 3-month (12-week) intervention.

The physical conditioning intervention was designed to serve as a rigorous time-and-attention control for the yoga intervention. Similarly, participants attended twice-weekly, 90-minute group classes for 12 weeks led by trainers or physical therapists who had received standardized training from the study physical therapy consultant (S.B.P.). Classes included upper and lower extremity stretching and strengthening exercises designed to be feasible for women across the aging spectrum but that avoided engaging the pelvic floor or promoting mindful relaxation. Participants were instructed to practice outside of class at least once a week during the intervention.

Group intervention instruction was initially delivered in person but transitioned to videoconference platforms after the COVID-19 pandemic social distancing mandates in March 2020.^20^ Thereafter, participants attended synchronous interventions classes delivered via videoconference platforms.

### Measurements

Sleep quality was assessed at baseline, 6 weeks, and the end of the 12-week intervention, as well as at another 12 and 24 weeks after the intervention using the Pittsburgh Sleep Quality Index (PSQI). This 19-item questionnaire assesses sleep quality, sleep onset latency, sleep efficiency, and additional sleep problems.^21^ The PSQI generates a score from 0 to 21, with scores greater than 5 indicating poor sleep quality, and has been validated across a variety of populations, including in women at midlife and older.^22,23,24^

Sleep duration and disruption were assessed at the same times using the Pittsburgh Sleep Diary, a validated self-report measure in which participants recorded their bedtimes, wake times, estimated time of sleep onset, and estimated middle of night awakenings over 3 days.^25^ Data were abstracted by blinded analysts to calculate the mean nightly total sleep time (TST), wakefulness after sleep onset (WASO; measured as minutes awake after initially falling asleep), and sleep efficiency (ratio of TST to total time in bed). Analysts also abstracted data to characterize reasons for nocturnal awakenings, including using the bathroom; noises, children, or bed partners; discomfort or a physical condition; or another reason.

Urinary symptoms were assessed using a validated 3-day voiding diary at baseline, 6 weeks, and 12 weeks of intervention, as well as 12 and 24 weeks after intervention instruction ended.^26^ Participants were instructed to record every episode of urine leakage or voiding in the toilet. Diary data were abstracted by blinded analysts who classified urinary symptoms by time of day, including nocturnal incontinence (leakage after going to bed for the night and before getting up in the morning) and nocturnal voiding (voiding in the toilet during this same time frame).

Participants self-reported baseline demographic characteristics, such as age, race (Asian, Black or African American, White, more than 1 race, or other) and ethnicity (Hispanic or Latina or not Hispanic or Latina), and educational background, as well as health-related behaviors. Participant-reported race and ethnicity data were collected to assess the generalizability of the study population and to adhere to National Institutes of Health policies. At a screening visit, clinical coordinators measured weight and height to determine body mass index (calculated as weight in kilograms divided by height in meters squared). Anxiety and depressive symptoms were additionally assessed by 2 self-report measures using the Hospital Anxiety and Depression Scale and Center for Epidemiology Studies Depression Scale.^27,28^ Clinical coordinators reviewed prescription and over-the-counter medications at baseline and intervention close-out.

### Statistical Analysis

Baseline participant characteristics in each group were examined using descriptive statistics. Mean baseline nocturnal incontinence and voiding frequency, as well as baseline levels of sleep outcome measures, were also examined in each group.

To evaluate intervention effects on global sleep quality (PSQI score), duration (TST), and disruption (WASO and sleep efficiency) as predefined secondary and ancillary trial end points, linear mixed models (LMMs) examined repeated changes in these outcomes from baseline to 6 and 12 weeks of intervention administration. Adjustment was done for baseline outcome levels, as well as 2 randomization stratification factors: study site and predominant baseline incontinence type. LMMs were run using Proc Mixed in SAS version 9.4 with fixed effects for visit, baseline incontinence type, study site, and baseline outcome value and nested subject within class random effects. Sleep outcome estimates were derived using least square means of change from baseline to 6 and 12 weeks and assuming approximately constant treatment effects over time. Additional models examined intervention effects specifically among participants with poor sleep quality at baseline (PSQI score >5).

Additional repeated-measure models assessed differences in mean sleep quality, TST, WASO, and sleep efficiency among participants with different levels of nocturnal incontinence or voiding frequency using data from baseline, 6-week, and 12-week times. In these models, data from both intervention groups were combined, but models adjusted for intervention assignment. Tests for linear trend assessed differences in sleep outcomes across participants categorized by nocturnal incontinence frequency (0, >0 to <1, or ≥1 episodes per night) and nocturnal voiding frequency (0, >0 to <1, 1 to <2, and ≥2 episodes per night).

Additional repeated-measure linear mixed models examined associations between prospective change in nocturnal voiding or incontinence frequency and prospective change in sleep outcomes from baseline to 6 and 12 weeks, again in a combined intervention group sample. These models adjusted for intervention assignment, study site, predominant incontinence type, and visit. All analyses were performed using SAS statistical software version 9.4 (SAS Institute). Data were analyzed from June to September 2024.

## Results

Between December 2018 and September 2022, a total of 240 participants were enrolled (mean [SD; range] age, 62.0 [8.7; 45-90] years; 40 Asian [21.1%], 19 Black [7.9%], 160 White [66.7%], and 20 more than 1 race [8.3%]; 32 Latina [13.3%]), including 121 individuals randomized to the yoga intervention and 119 individuals randomized to the physical conditioning intervention. The mean (SD) incontinence frequency was 3.4 (2.2) episodes per day. Of participants randomized, 105 individuals (87.6%) completed the 12-week yoga intervention and 108 individuals (90.8%) completed the 12-week physical conditioning intervention (ie, were still engaged in interventions at 12 weeks) (Figure). A total of 76 participants received all in-person instruction, while 164 participants received primarily videoconference-based instruction. Among 120 retained yoga participants and 119 physical conditioning participants, 100 participants (93.4%) and 98 participants (89.1%), respectively, completed more than 80% of sessions. As previously reported, incontinence frequency decreased by 2.3 episodes per day in the yoga group and 1.9 episodes per day in the physical conditioning group over 12 weeks, but between-group differences did not meet the trial’s prespecified significance threshold.^19^

**Figure.**
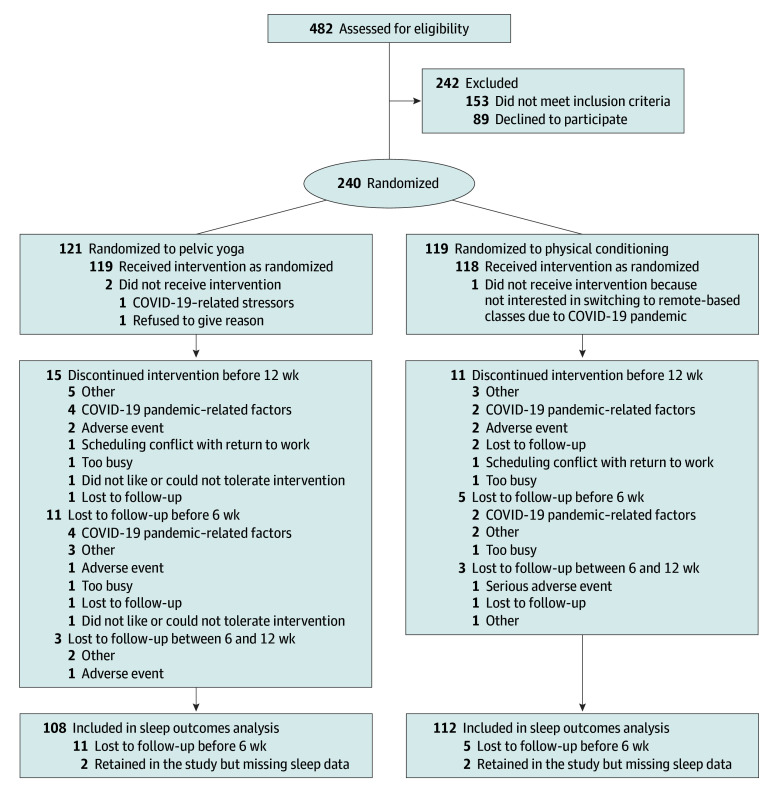
Study Flowchart

Baseline characteristics were similar across the 2 groups (Table 1), although antidepressant use was higher in the physical conditioning (22 participants [18.5%]) than in the yoga (10 participants [8.3%]) group. More than 55% of participants had a PSQI score greater than 5 (133 participants [55.4%]), indicating impaired baseline sleep quality.

**Table 1.** Baseline Participant Clinical and Demographic Characteristics

Characteristic	Participants, No. (%) (N = 240)^a^	
	Yoga therapy (n = 121)	Physical conditioning (n = 119)
Age, y		
Mean (SD)	62.7 (8.7)	61.4 (8.8)
Median (range)	63.0 (45.0-90.0)	62.0 (46.0-80.0)
Race		
Asian	20 (16.5)	20 (16.8)
Black or African American	7 (5.8)	12 (10.1)
White	83 (68.6)	77 (64.7)
More than 1 race	11 (9.1)	9 (7.6)
Other^b^	0	1 (0.8)
Ethnicity		
Hispanic or Latina	21 (17.4)	11 (9.2)
Not Hispanic or Latina	99 (81.8)	108 (90.8)
Unknown	1 (0.8)	0
Education		
≥College degree	98 (81.0)	101 (84.9)
≤High school	5 (4.1)	5 (4.2)
Some college or AA degree	18 (14.9)	13 (10.9)
Chronic health conditions		
Arthritis	40 (33.1)	32 (26.9)
Respiratory disease	21 (17.4)	19 (16.0)
Diabetes	8 (6.6)	9 (7.6)
Heart disease	6 (5.0)	2 (1.7)
Health-related habits		
Former cigarette smoking	29 (24.0)	25 (21.0)
Current cigarette smoking	0	1 (0.8)
Current weekly alcohol consumption	57 (47.1)	52 (43.7)
Physical activity, mean (SD), METs/week	2270 (2354)	1896 (1739)
BMI		
No. with data	121	118
Mean (SD)	27.38 (5.1)	27.11 (5.7)
Selected medications		
Benzodiazepine	2 (1.7)	2 (1.7)
Anticonvulsant	3 (2.5)	5 (4.2)
Melatonin	3 (2.5)	2 (1.7)
Nonbenzodiazepine	0	1 (0.8)
Opioid	1 (0.8)	0
Antidepressant	10 (8.3)	22 (18.5)
Antipsychotic	0	1 (0.8)
Antihistamine	16 (13.2)	22 (18.5)
Predominant incontinence type		
Stress or stress predominant	47 (38.8)	47 (39.5)
Urgency or urgency predominant	74 (61.2)	72 (60.5)
Incontinence frequency, episodes/d, mean (SD)		
Any or total incontinence	3.6 (2.5)	3.2 (1.9)
Urgency incontinence	2.1 (2.2)	1.7 (1.4)
Stress incontinence	1.4 (1.8)	1.4 (1.5)
Duration of incontinence, y		
<1	5 (4.1)	6 (5.0)
1 to 4	44 (36.4)	40 (33.6)
≥5	72 (59.5)	73 (61.3)
Nighttime incontinence, episodes per night		
Mean (SD)	0.4 (0.7)	0.2 (0.3)
0	72 (59.5)	77 (64.7)
>0 to <1	30 (24.8)	34 (28.6)
1 to <2	12 (9.9)	8 (6.7)
2 to <3	5 (4.1)	0
3 to <4	2 (1.7)	0
≥4	0	0
Nighttime voiding, episodes/night		
Mean (SD)	1.1 (1.0)	0.9 (0.8)
0	27 (22.3)	26 (21.8)
>0 to <1	37 (30.6)	39 (32.8)
1 to <2	34 (28.1)	38 (31.9)
2 to <3	11 (9.1)	14 (11.8)
3 to <4	11 (9.1)	2 (1.7)
≥4	1 (0.8)	0
Sleep outcomes		
PSQI global score		
No. with data	118	118
Mean (SD)	6.2 (3.1)	6.6 (3.1)
Mean (SD) if >5	8.54 (2.3)	8.99 (2.3)
≤5	48 (40.7)	55 (46.6)
>5 to <9 (Mild sleep problems)	41 (34.7)	38 (32.2)
≥9 (Moderate to severe sleep problems)	29 (24.6)	25 (21.2)
TST		
No. with data	120	119
Mean (SD), min	422.4 (70.5)	419.5 (60.5)
WASO		
No. with data	120	117
Mean (SD), min	25.0 (29.7)	25.0 (25.3)
Sleep efficiency		
No. with data	120	119
Mean (SD), %	83 (10)	82 (10)

Abbreviations: AA, associate in arts; BMI, body mass index (calculated as weight in kilograms divided by height in meters squared); MET, metabolic equivalent of task; PSQI, Pittsburgh Sleep Quality Index; TST, total sleep time; WASO, wake after sleep onset.

^a^ Data are missing for 1 participant for BMI, 4 participants for PSQI, 1 participant for TST, 3 participants for WASO, and 1 participant for sleep efficiency.

^b^ One participant selected *Other* as the response option for the study question inviting her to self-report race; this participant further indicated that she was Hispanic.

On baseline sleep diaries, 133 participants of 239 participants with data (55.7%) reported awakening at least once per night to use the bathroom, with 46 participants (19.3%) reporting at least 2 awakenings to use the bathroom. Other self-reported causes of awakenings, such as noises, child, or partner; pain; or other reason or no reason, were less commonly cited (Table 2).

**Table 2.** Participants Reporting Nocturnal Awakenings at Baseline (N = 239)

Reason for awakening	Awakenings per night, No. %, mean (SD) of 3 diary days (N = 239)^a^				
	0	0.1-0.9	1.0-1.9	2.0-2.9	≥3
Awakenings for any reason at night	13 (5.4)	34 (14.2)	71 (29.7)	67 (28.0)	54 (22.6)
Awakenings to go to bathroom	43 (18.0)	63 (26.4)	87 (36.4)	31 (13.0)	15 (6.3)
Awakenings due to noises, child, or partner	143 (59.8)	58 (24.3)	29 (12.1)	8 (3.35)	1 (0.4)
Awakenings due to pain	176 (73.6)	45 (18.8)	14 (5.9)	4 (1.7)	0
Awakenings due to other or no reason	139 (58.2)	64 (26.8)	22 (9.2)	8 (3.4)	6 (2.5)

^a^ Percentages are row percentages; all denominators are the 239 participants with responses to this question.

Of 240 randomized participants, 220 participants provided follow-up sleep outcomes data at 6 and 12 weeks. The 20 participants missing follow-up sleep data compared with retained participants were older (mean [SD] age, 66.5 [7.4] years vs 61.7 [8.7] years; *P* = .02) and reported predominantly urge incontinence (17 participants [85.0%] vs 129 participants [58.6%]; *P* = .03) rather than stress-predominant incontinence but did not differ in other general health or sleep characteristics. No clinically important differences in the effects of the 2 intervention programs on sleep outcomes were detected from baseline to 6 and 12 weeks among analyses involving all participants. Compared with the physical conditioning group (mean change, 0.66 points [95% CI, 1.07 to 0.25 points]), the yoga intervention (mean change, 0.37 points [95% CI, 0.78 to −0.04 points]) had a nonsignificant mean change of 0.29 points (95% CI, −0.28 to 0.86 points) in PSQI global score. Similarly, in WASO, the yoga group (mean change, 3.82 minutes [95% CI, 8.15 to −0.52 minutes]) compared with the physical conditioning group (mean change, 6.97 minutes [95% CI, 11.20 to 2.74 minutes]) did not have a significant difference (mean difference, 3.16 minutes [95% CI, −2.84 to 9.16 minutes]). Mean changes comparing yoga with physical conditioning were also nonsignificant for TST (−3.80 minutes [95% CI, −18.60 to 10.99 minutes]) and sleep efficiency (−1.46% [95% CI, −3.65% to 0.73%]) in sleep efficiency (Table 3). Additionally, no statistically significant within-group improvements in PSQI global sleep quality score, TST, WASO, or sleep efficiency were detected among participants assigned to the yoga intervention. However, participants in the physical conditioning group demonstrated a mean decrease of 0.66 points (95% CI, 1.07 to 0.25 points) in global PSQI score, a mean improvement of 6.97 minutes (95% CI, 11.20 to 2.74 minutes) in WASO, and a mean improvement of 2.40% (95% CI, 0.87% to 3.94%) in sleep efficiency (Table 3).

**Table 3.** Change in Sleep Outcomes

Intervention group	Participants, No.	Change from baseline to 6 wk and 12 wk, mean (95% CI)^a^		
		Yoga therapy	Physical conditioning	Between-group difference
All available randomized participants				
PSQI global score (0-21), points	220	−0.37 (−0.78 to 0.04)	−0.66 (−1.07 to −0.25)	0.29 (−0.28 to 0.86)
TST, min	217	−0.12 (−10.90 to 10.65)	3.69 (−6.69 to 14.06)	−3.80 (−18.60 to 10.99)
WASO, min^b^	216	−3.82 (−8.15 to 0.52)	−6.97.0 (−11.20 to −2.74)	3.16 (−2.84 to 9.16)
Sleep efficiency, %^b^	217	0.94 (−0.65 to 2.53)	2.40 (0.87 to 3.94)	−1.46 (−3.65 to 0.73)
Participants with baseline PSQI score >5				
PSQI global score (0-21), points	126	−0.91 (−1.55 to −0.27)	−1.27 (−1.88 to −0.66)	0.36 (−0.50 to 1.22)
TST, min	126	−6.98 (−22.40 to 8.46)	−1.23 (−15.80 to 13.36)	−5.75 (−26.60 to 15.09)
WASO, min^b^	125	−6.41 (−13.20 to 0.39)	−9.04 (−15.60 to −2.45)	2.63 (−6.69 to 11.95)
Sleep efficiency, %^b^	126	0.32 (−1.95.0 to 2.59)	3.29 (1.14 to 5.43)	−2.96 (−6.03 to 0.10)

Abbreviations: PSQI, Pittsburgh Sleep Quality Index; TST, total sleep time; WASO, wake after sleep onset.

^a^ Estimates of mean change values and 95% CIs are derived from linear mixed models including both 6- and 12-week times adjusted for baseline values, study site, and predominant clinical incontinence type as original randomization stratification factors; models also accounted for potential clustering by intervention class and cohort. Models include participants with follow-up data for each outcome at either 6 or 12 weeks; values are least square mean estimates of change at both follow-up times.

^b^ Sleep efficiency and WASO data were winsorized to address skewed data distributions.

Similarly, among the subset of 133 participants with global PSQI score greater than 5 at baseline, no significant between-group differences in change in sleep outcomes from baseline to 6 and 12 weeks were observed. However, within-group improvements in PSQI score were observed in both groups, with yoga participants reporting a mean decrease of 0.91 points (95% C,I 1.55-0.27 points) and physical conditioning participants reporting an mean decrease of 1.27 points (95% CI, 1.88-0.66 points) from baseline to 6 and 12 weeks. Within the physical conditioning group, improvements in WASO and sleep efficiency were also detected (Table 3).

In additional models combining data from intervention groups and all time points, greater nocturnal incontinence frequency was associated with worse sleep efficiency and increased disruption. For example, mean sleep efficiency was 83.30% (95% CI, 81.91%-84.71%) among participants reporting a mean of no incontinence episodes per night, 80.06% (95% CI, 77.92%-82.26%) among those reporting a mean of more than 0 to less than 1 incontinence episode per night, and 78.49% (95% CI, 75.19%-81.94%) among those reporting a mean of at least 1 incontinence episode per night (*P* = .008) (Table 4). Greater nocturnal voiding frequency was associated with worse sleep quality, disruption, and efficiency. For example, the mean global PSQI score was 5.83 points (95% CI, 5.35-6.32 points) for participants with a mean of no voids per night, 5.82 points (95% CI, 5.38-6.25 points) in participants with a mean of less than 1 void per night, 6.14 points (95% CI, 5.68-6.60 points) in those with a mean of at least 1 to less than 2 voids per night, and 6.66 points (95% CI, 6.00-7.33 points) in those with a mean of at least 2 voids per night (*P* for linear trend = .02).^29^

**Table 4.** Mean Sleep Outcomes by Nocturnal Urinary Symptom Frequency

Sleep outcome	**Participants, No.**	Mean (95% CI)^a^				*P *for trend^b^
		Frequency group 1	Frequency group 2	Frequency group 3	Frequency group 4	
**By mean nocturnal incontinence frequency** ^c^						
PSQI global score (0-21), points	236	5.94 (5.56-6.31)	6.12 (5.59-6.65)	6.60 (5.81-7.40)	NA	.09
TST, min	240	423.00 (415.55-430.45)	420.28 (408.15-432.41)	416.34 (396.76-435.91)	NA	.51
WASO, min^d^	239	8.51 (7.01-10.32)	14.93 (10.74-19.29)	16.20 (10.17-25.82)	NA	.007
Sleep efficiency, %^d^	240	83.30 (81.91-84.71)	80.06 (77.92-82.26)	78.49 (75.19-81.94)	NA	.008
**By mean nocturnal voiding frequency^e^**						
PSQI global score (0-21), points	236	5.83 (5.35-6.32)	5.82 (5.38-6.25)	6.14 (5.68-6.60)	6.66 (6.00-7.33)	.02
TST, min	240	419.37 (408.49-430.25)	413.37 (404.01-422.73)	432.78 (422.79-442.78)	425.17 (410.06-440.29)	.20
WASO, min^d^	239	4.79 (3.69-6.21)	9.03 (7.18-11.35)	14.60 (11.44-18.65)	19.04 (13.23-27.40)	<.001
Sleep efficiency, %^d^	240	84.55 (82.48-86.67)	82.65 (80.90-84.45)	82.23 (80.36-84.13)	77.89 (75.22-80.66)	<.001

Abbreviations: PSQI, Pittsburgh Sleep Quality Index; TST, total sleep time; WASO, wake after sleep onset.

^a^ Data are adjusted for intervention group, clinical site, and predominant urinary incontinence type and given by mean nocturnal incontinence or voiding frequency.

^b^ *P* values were calculated for tests of linear trend in sleep parameters across increasing categories of nocturnal incontinence or voiding frequency.

^c^ For incontinence, mean frequency groups were 0 incontinence episodes per night (group 1), more than 0 to less than 1 incontinence episodes per night (group 2), and 1 or more incontinence episodes per night (group 3), with no group 4.

^d^ Sleep efficiency and WASO data were natural log transformed to address skewed data distribution.

^e^ For voiding, mean frequency groups were 0 voids per night (group 1), more than 0 to less than 1 voids per night (group 2), 1 to less than 2 voids per night (group 3), and 2 or more voids per night (group 4).

However, in analyses assessing associations between prospective change in frequency of nighttime urinary symptoms and prospective change in sleep outcomes, the magnitude of improvement in nocturnal voiding or incontinence frequency was not correlated with the magnitude of improvement in any sleep outcome (eTable in Supplement 2). As previously reported,^19^ 92 adverse events were detected, without significant differences between groups.

## Discussion

In this secondary analysis of a multicenter randomized clinical trial in women at midlife and older with urinary incontinence, participants assigned to a therapeutic yoga intervention did not demonstrate superior sleep outcomes compared with those assigned to a general physical conditioning intervention. Sleep quality, disruption, and efficiency did not differ significantly between groups, although participants in both groups who experienced poor sleep quality at baseline reported improvements in sleep outcomes over 3 months. Despite increasing interest in yoga as a nonpharmacologic intervention for sleep, our results do not confirm that yoga offers unique benefits for sleep above and beyond other physical movement–based activities.

These findings may seem surprising given that a 2020 meta-analysis^18^ reported that engagement in yoga was associated with sleep outcome improvements. However, past studies reporting benefits of yoga for sleep have featured variable study designs, types of yoga interventions and comparators, and participant characteristics. Most studies compared yoga with no active treatment, making it difficult to determine whether yoga had a unique and specific impact on sleep or whether improved sleep could also have been realized through other forms of physical activity.

To date, yoga’s effects on sleep have been studied in populations with different chronic conditions and syndromes, including cancer,^30,31,32^ osteoarthritis,^33^ and menopausal symptoms, with mixed results.^34,35^ Our study expands this body of research by providing unique data on sleep quality, disruption, and efficiency among a representative population of women at midlife and older with incontinence who were engaging with yoga. Notably, many participants in this trial reported poor sleep quality at baseline, with 55.7% having a PSQI score greater than 5, suggesting clinically significant sleep problems. Furthermore, at any given time, participants who reported frequent nocturnal urination also reported more substantial sleep disruption and worse sleep quality and efficiency. Thus, our results reinforce known associations between poor sleep and disruptive nocturnal urinary symptoms, such as nocturia.^36,37^

Interestingly, improvement in nocturnal urinary symptoms did not necessarily translate into improvement in sleep outcomes. Although participant mean sleep quality improved from baseline to 3 months, the magnitude of their improvement in sleep quality did not correlate with the magnitude of their improvement in nocturnal urinary symptoms. These results underscore that sleep disruption can be multifactorial; even if nocturnal urinary symptoms improve, sleep problems may persist for other reasons.

### Limitations

Several limitations of this study should be acknowledged. Sleep was assessed using self-reported diaries and questionnaires, without corroboration by physiologic measures. Future research should assess accompanying changes in actigraphy or polysomnography measures. Additionally, while sleep outcomes were prespecified secondary outcomes of the trial, the yoga intervention was not specifically designed as a sleep intervention, and not all participants had poor sleep at baseline.^38^ Therefore, findings could differ for other types of yoga interventions or in populations with uniformly impaired sleep. The trial was also affected by the COVID-19 pandemic, which required a shift from in-person to primarily remote intervention instruction. Therefore, our results may not be generalizable to adults learning to practice yoga in person. In addition to class format limitations, our results could be limited by the frequency of intervention delivery. Classes occurred twice weekly, with at least 1 hour of additional self-directed practice per week; therefore, the effects of yoga on sleep may be greater with more frequent practice. Nonetheless, variability of practice rates across previous studies ranges from once weekly to 3 times per week.^34,35,39,40^ In addition, the trial design compared 2 active interventions, which allowed us to evaluate the unique effects of yoga on sleep; however, we cannot draw direct conclusions about whether the practice of yoga offered benefits for sleep compared with no physical activity.

## Conclusions

Findings from this secondary analysis of a randomized clinical trial provide new evidence regarding the effects of a therapeutic yoga intervention in older women with urinary incontinence as a common chronic condition. As interest in complementary therapies like yoga increases, evaluation of their effects on sleep through well-designed clinical trials is important for guiding physician-treatment recommendations. While many people who engage in yoga practice perceive a positive impact on their sleep, findings from this study do not indicate the superiority of yoga to general exercise for promoting sleep.

## Notes

Supplement 1.
Trial Protocol and Statistical Analysis Plan

Supplement 2. **eTable.** Prospective associations between change in nocturnal urinary incontinence and nocturnal voiding and change in sleep outcomes from baseline to 6 and 12 weeks in the combined-group sample

Supplement 3.
Data Sharing Statement
