# Supplementary material for: Effects of Hatha Yoga vs Physical Conditioning on Sleep in Women With Urinary Incontinence: A Secondary Analysis of a Randomized Clinical Trial
Source: JAMA Netw Open. 2025 Dec 8;8(12):e2546499. doi: 10.1001/jamanetworkopen.2025.46499 (PMC12687092; doi:10.1001/jamanetworkopen.2025.46499)
Supplement: Supplement 1. — Trial Protocol and Statistical Analysis Plan [file jamanetwopen-e2546499-s001.pdf]

A Randomized Controlled Trial of a Group-Based Therapeutic Yoga Intervention for Urinary Incontinence in Ambulatory Older Women

NCT03672461

Version Date: 02/27/2023

IRB Approval Date: 03/10/2023

Lessening Incontinence through Low-impact Activity (LILA)

(a.k.a. A Randomized Controlled Trial of a Group-Based Therapeutic Yoga Intervention for Urinary Incontinence in Ambulatory Older Women)

Principal investigator:

Alison Huang, MD, MAS
Associate Professor of Medicine, Urology, and Epidemiology and Biostatistics
University of California San Francisco

Supported by:

The National Institute of Diabetes, Digestive, and Kidney Disorders
Grant #1R01DK116712-01A1

Protocol Revision History

Version Number: 1.0
Version Date: 10/18/2018

Version Number: 2.0
Version Date: 11/16/2018

- Section 4.2: Clarified Exclusion Criteria
- Section 5.2: Added National Strength and Conditioning Association an approved society for certification
- Section 6.15: Replaced SPPB with Balance Testing and 30 second chair stand.
- Section 6.18: added Fluid Intake Questionnaire
- Section 6.25: Added 9-Week Phone Call description

Version Number: 3.0
Version Date: 01/24/2019

- Section 6.1.7a: Perineometer measurements
- Section 7.1: added Potential Risks of Perineometer measurement

Version Number: 4.0
Version Date: 02/25/2019

- Section 5.2: discovered the National Strength and Conditioning Association (NSCA) was not added within the text of the protocol, although this was listed above.

Version Number: 5.0
Version Date: 06/11/2019

- Sections: 6.1.8 Additional Screening and Covariate Measures and 6.2.7 Study Measurements Table: discovered that blood pressure and weight measurements were incorrectly indicated at week 6. Therefore, we are correcting the protocol to correctly reflect the approved informed consent document.

Version Number: 6.0
Version Date: 10/02/2019

- Section: 6.2.5 Follow-up/Final Evaluations: We discovered a typo in the visit window for 36 weeks. The text should state “Within 273 days (39 weeks)”, rather than “Within 252 days (39 weeks)”. Therefore, the protocol has been corrected to include the correct number of days for 39 weeks.

Version Number: 7.0
Version Date: 6/12/2020

- Sections 5.1 and 5.2 added the possibility of remote class instruction during COVID Pandemic
- Sections 6.16 and 6.17: During the COVID-19 pandemic, participants may decline measurements due to close proximity with study personnel.
- Section 9.6.2 Due to COVID 19 Pandemic, we may utilize subgroup analyses for to evaluate differences in remote instruction and in-person instruction.

Version Number 8.0
Version Date: 7/14/2021

Sections 3.0 and 9.2 have been updated to 250 participants randomized, with about 125 participants per group, recognizing that baseline frequency of urinary incontinence may be closer to 22-23 rather than 25 episodes per week.

Sections 6.25 has been updated to indicate that selected participants in the yoga arm may be invited to take part in qualitative interviews after the 12-Week Visit to discussed perceived barriers and facilitators to learning to practice yoga.

Version Number 9.0
Version Date: 02/27/2023

Sections 3.0 and 9.2 have been updated to 240 participants randomized, with about 120 participants per group, recognizing that baseline frequency of urinary incontinence may be closer to 23-24 rather than ~22-23 episodes per week.

TABLE OF CONTENTS

PRÉCIS 8
1. STUDY OBJECTIVES 9
2. BACKGROUND AND RATIONALE 10
2.1 Background on Condition 10
2.2 Study Rationale 10
3. STUDY DESIGN 11
4. SELECTION AND ENROLLMENT OF PARTICIPANTS 12
4.1 Inclusion Criteria 12
4.2 Exclusion Criteria 12
4.3 Study Enrollment Procedures 13
5. STUDY INTERVENTIONS 14
5.1 Yoga Program 14
5.2 Muscle Stretching/Strengthening (i.e., Physical Conditioning) Control Program 16
5.3 Concomitant Interventions 18
5.3.1 Required Interventions 18
5.3.2 Prohibited or Discouraged Interventions 18
5.4 Adherence Assessment 19
6. STUDY PROCEDURES 20
6.1 Study Measures 20
6.1.1 Urinary Incontinence 20
6.1.2 Incontinence-Related Quality of Life 20
6.1.3 Depression, Anxiety, and Perceived Stress 20
6.1.4 Yoga/Physical Conditioning Self-efficacy and Competency 21
6.1.5 Physical Function/Performance Measures 22
6.1.6 Cardiac Autonomic Function Measures 22
6.1.7 Pelvic Floor Muscle Strength 23
6.1.8 Additional Screening and Covariate Measures 24
6.2 Evaluations 24
6.2.1 Screening Evaluations 24
6.2.2 Consenting Procedure 25
6.2.3 Screening Procedures 25
6.2.4 Randomization and Blinding 26
6.2.5 Follow-up/Final Evaluations 28
6.2.6 Early Termination (also see section below) 31
6.2.7 Table of Measures and Procedures 32
7. SAFETY ASSESSMENTS 33
7.1 Potential Risks and Protective Measures 33
7.2 Methods and Timing of Safety Assessments 35
7.3 Adverse Events and Serious Adverse Events 35
7.3.1 AE/SAE Definitions 35
7.3.2 AE/SAE Documentation 35
7.4 Reporting Procedures 36
7.4.1 SAE Reporting 36
7.4.2 Non-Serious AE Reporting 36
7.5 Follow-up for AEs/SAEs 37
7.6 Independent Safety Monitoring 37
8. INTERVENTION DISCONTINUATION 38
9. STATISTICAL CONSIDERATIONS 38

9.1 General Design Issues 38
9.2 Sample Size 39
9.3 Randomization and Blinding 39
9.4 Interim Analyses and Stopping Guidelines 40
9.5 Outcomes 40
9.5.1 Primary Efficacy Outcome 40
9.5.2 Secondary Efficacy Outcomes 40
9.6 Data Analyses 41
9.6.1 Analyses to address main objectives 41
9.6.2 Other analytic issues 43
10. DATA COLLECTION AND QUALITY ASSURANCE 44
10.1 Data Collection Forms 44
10.2 Data Management 45
10.3 Quality Assurance 45
10.3.1 Staff Training 45
10.3.2 Data Quality Assessment 46
10.3.3 Protocol Deviations 46
10.3.4 External Monitoring 46
11. PARTICIPANT RIGHTS AND CONFIDENTIALITY 47
11.1 Institutional Review Board (IRB) Review 47
11.2 Informed Consent Forms 47
11.3 Participant Confidentiality 47
11.4 Study Discontinuation 47
12. ETHICAL CONSIDERATIONS 47
13. STEERING/PUBLICATIONS COMMITTEE 47
14. PUBLICATION OF RESEARCH FINDINGS 48
15. REFERENCES 48

STUDY TEAM ROSTER

Investigators	
Alison Huang, MD, MAS	1545 Divisadero Street, Suite 322<br>San Francisco, CA 94115<br>E-mail: ahuang@medicine.ucsf.edu<br>Tel: 415-514-8697 F: 415-514-8666
Leslee Subak, MD	300 Pasteur Drive, HG332, Office #G-303A<br>Stanford, CA 94305-5317<br>E-mail: lsubak@stanford.edu<br>Tel: 650-723-5533 F: 650-723-7737
Margaret Chesney, PhD	1545 Divisadero Street, Suite 508<br>San Francisco, CA 94115<br>Email: chesneym@ocim.ucsf.edu<br>Tel: 415-353-7719 F: 415-353-7711
Wendy Mendes, PhD	3333 California Street<br>San Francisco, CA 94118<br>Email: Wendy.Mendes@ucsf.edu<br>Tel: 415-476-7409
Eric Vittinghoff, PhD	550 16th Street, 3rd Floor<br>San Francisco, CA 94158<br>Email: Eric.Vittinghoff@ucsf.edu<br>Tel: 415-514-8025
Project Directors	
Ann Chang	550 16th Street, 6th Floor<br>San Francisco, CA 94158<br>Email: Ann.Chang@ucsf.edu<br>Tel: 415-353-9782 F: 415-476-5367
Lisa Abinanti	550 16th Street, 6th Floor<br>San Francisco, CA 94158<br>Email: Lisa.Abinanti@ucsf.edu<br>Tel: 415-353-9978 F: 415-476-5367
Data Management and Statistical Analysts	
Michael Schembri	550 16th Street, 6th Floor<br>San Francisco, CA 94158<br>Email: michael.schembri@ucsf.edu<br>Tel: 415-353-7161 F: 415-476-5367
Data and Safety Monitor	
Andrew Avins, MD, MPH	Kaiser Permanente Division of Research<br>2000 Broadway<br>Oakland, CA 94612<br>Email: andy.l.avins@kp.org<br>Tel: (510) 891-3557 (phone)

PRÉCIS

Study Title

Lessening Incontinence through Low-impact Activity (LILA), a.k.a. A Randomized Controlled Trial of a Group-Based Therapeutic Yoga Intervention for Urinary Incontinence in Ambulatory Older Women

Objectives

To evaluate the efficacy of a group-based yoga intervention for urinary incontinence in ambulatory middle-aged and older women and explore potential mediators of intervention effects in this population.

Study Design

Randomized, multicenter, parallel-group trial of a 12-week yoga intervention versus non-specific muscle stretching/strengthening control intervention for ambulatory women aged 45 years and older with urinary incontinence.

Sample Size and Population

Two hundred fifty women aged 45 years and older who document stress-, urgency-, or mixed-type incontinence occurring at least once per day, are not participating in other organized yoga or muscle stretching/strengthening activities, are temporarily willing to forgo other established clinical treatments for incontinence, and meet minimum mobility and other clinical eligibility criteria, recruited from multiple locations in northern California.

Interventions and Duration

Participants will be randomized in a 1:1 ratio to participate in either a 12-week yoga program (N~120) or a time-equivalent non-specific muscle stretching/strengthening control program (N~120). Women randomized to the yoga program will take part in twice weekly group yoga classes focusing on Iyengar/alignment-based yoga techniques and practice yoga at home for at least one hour per week for 12 weeks. Women randomized to the control program will take part in twice weekly group classes focusing on non-specific muscle stretching/strengthening exercises and practice these exercises at least one hour per week at home for 12 weeks.

Study Outcomes

The primary outcome is change in the frequency of any urinary incontinence episodes between baseline and 12 weeks, measured using validated voiding diaries. Secondary outcomes include changes in the frequency of stress-type, urgency-type, daytime, and nighttime incontinence episodes (also measured by voiding diaries), as well as changes in incontinence-related functioning or quality of life (measured by validated questionnaires), between baseline and 12 weeks. Additional secondary outcomes include persistence of changes in incontinence frequency and condition-specific quality of life 12 and 24 weeks after the end of the intervention programs.

1. STUDY OBJECTIVES

Objective 1: To determine the efficacy of a group-based yoga intervention in reducing the frequency of urinary incontinence in ambulatory midlife and older women.

Hypothesis 1A: Ambulatory incontinent women randomized to the yoga intervention will demonstrate at least a 20% greater decrease in total incontinence frequency (measured by validated voiding diaries) over 12 weeks, compared to women randomized to the control intervention.

Hypothesis 1B: If improvements in total incontinence frequency are observed at 12 weeks, these improvements will be sustained 12 and 24 weeks after the end of the intervention programs.

Objective 2: To determine the efficacy of a group-based yoga intervention in reducing the frequency of type- and context-specific urinary incontinence in ambulatory middle-aged and older women.

Hypothesis 2A: Ambulatory incontinent women randomized to the yoga intervention will report decreases in the frequency of stress-type, urgency-type, daytime, and nighttime incontinence (also measured by voiding diaries) over 12 weeks, compared to women randomized to the control intervention.

Hypothesis 2B: If improvements in stress-type, urgency-type, daytime, and nightime incontinence frequency are observed at 12 weeks, these improvements will be sustained 12 and 24 weeks after the end of the intervention programs.

Objective 3: To determine the efficacy of the yoga intervention in improving incontinence-related functioning and quality-of-life.

Hypothesis 3A: Ambulatory incontinent women randomized to the yoga intervention will report greater improvements in condition-specific functioning and quality of life (assessed by validated questionnaires) over 12 weeks, compared to women randomized to the control intervention.

Hypothesis 3B: Compared to women randomized to the control intervention, we hypothesize that women randomized to the yoga intervention will report sustained improvements in condition-specific quality of life 12 and 24 weeks after the end of the intervention programs.

Objective 4: To explore whether the yoga intervention is associated with improvements in 1) pelvic floor muscle strength, 2) peripheral autonomic function, and/or 3) lower extremity physical function, and assess whether improvements in these factors may mediate improvement in incontinence.

Hypothesis 4A: Among ambulatory incontinent women enrolled in this trial, the yoga intervention will be associated with improvements in a) pelvic floor muscle strength (assessed by pelvic exam), b) cardiac autonomic function (assessed by heart rate variability and impedance cardiography), and c) lower extremity physical function (assessed by physical performance assessment) over 12 weeks.

Hypothesis 4B: Among ambulatory incontinent women enrolled in this trial, changes in incontinence frequency over 12 weeks will be partly mediated by changes in a) pelvic floor muscle strength, b) cardiac autonomic function, and c) lower extremity physical function.

2. BACKGROUND AND RATIONALE

2.1 Background on Condition

Approximately one in four midlife and older women suffers from urinary incontinence, a condition associated with depression, social isolation, functional decline, falls and fractures, and admission to long-term care facilities.[1-8] In economic analyses, the annual estimated direct costs of incontinence among U.S. women exceed $20 billion, greater than that of breast, ovarian, cervical, and uterine cancers combined.[9]

Current first-line treatment for incontinence includes behavioral management strategies such as bladder re-training and pelvic floor exercises designed to increase bladder capacity, strengthen the pelvic floor muscles supporting the bladder, and suppress involuntary bladder contractions. Unfortunately, many patients who attempt to practice these techniques after routine teaching by general practitioners are not able to do so effectively.[10-13] Although the efficacy of these techniques can be improved by one-on-one training and biofeedback with pelvic physical therapists or other specialized medical practitioners, this type of intensive pelvic floor rehabilitation therapy is costly, and access to trained pelvic physical therapists is limited.[14,15]

Second-line treatment for urgency-type incontinence (i.e., urine leakage caused by sudden or strong urges to urinate) consists primarily of anticholinergic drugs that are modestly effective in reducing incontinence, but have multiple side effects such as dry mouth, stomach upset, constipation, and even cognitive impairment. As a result, over half of patients who initiate anticholinergic therapy for urinary symptoms like incontinence discontinue it within a year.[16-18] For stress-type incontinence (i.e., urine leakage with activities that increase abdominal pressure), surgery and other invasive procedures can be effective, but are poorly tolerated by many women, particularly older or frailer women who are at greatest risk of developing frequent incontinence.[19] As a result, there is a need for alternate treatment strategies that that are not only effective, but also more accessible and better tolerated.

2.2 Study Rationale

Yoga is a set of complementary physical and mental practices with the potential to improve urinary incontinence through multiple mechanisms, while also avoiding many of the shortcomings of existing clinical therapies. When taught in a way that emphasizes mindful awareness of specific bodily structures, yoga can be used to help women control and strengthen their pelvic floor muscles through a combination of group instruction and home practice. As a result, a group-based yoga program that incorporates practice of specific yoga postures to promote awareness and engagement of the pelvic floor may provide a more accessible alternative to traditional, one-on-one pelvic floor rehabilitation therapy.

Yogic breathing and relaxation techniques also have the potential to improve peripheral autonomic dysfunction as another potential contributor to incontinence. Prior physiologic studies have demonstrated that many women with urgency-type incontinence have to have abnormalities in sympathetic and parasympathetic autonomic function.[20-25] Yoga interventions that involve regular practice of yogic breathing have the potential to improve resting autonomic function as well as stress-related autonomic reactivity,[26-29] which could in turn lead to improved bladder control.

Regular practice of yoga postures can also improve physical deconditioning as another

potential contributor to incontinence risk.[5,30-33] Among frail older women, decreased physical conditioning and mobility can compromise women's ability to get to the bathroom when they experience urges to urinate. Poor physical conditioning is also associated with increased abdominal obesity, which has been shown to contribute to both stress- and urgency-type incontinence symptoms. Several small studies have explored the benefits of physical exercise programs for preventing or managing incontinence in older women[34,35]; unlike yoga, however, these non-specific exercise programs did not have the added benefit of engaging the pelvic floor or directly improving autonomic balance in incontinent women.

To examine the feasibility of using yoga to treat incontinence in middle-aged and older women, our research team previously developed a group-based, Iyengar-style yoga program consisting of twice weekly classes and once weekly home practice sessions. We subsequently pilot-tested and refined that program through an initial pilot randomized trial involving 19 women and a follow-up feasibility trial involving 56 women. We will now conduct a rigorous multicenter randomized trial to determine whether the refined group-based yoga intervention is effective in decreasing the frequency and impact of urinary incontinence in middle-aged and older women and explore potential mediators of intervention effects in this population.

3. STUDY DESIGN

This is a randomized, multicenter, parallel-group trial to determine whether a 12-week group-based yoga intervention is effective in decreasing the frequency and impact of urinary incontinence in middle-aged and older women. Women aged 45 years and older who document at least daily episodes of stress-, urgency-, or mixed-type incontinence on a screening voiding diary, are not using other clinical treatments for incontinence, and meet minimum physical mobility requirements and other eligibility criteria will be recruited from multiple locations in northern California by coordinators affiliated with the University of California San Francisco or Stanford University. Eligible women will be randomized in a 1:1 ratio to participate in either a 12-week yoga program (N~120) or a time-equivalent, non-specific muscle stretching and strengthening control program (N~120).

Women randomized to the yoga program will take part in twice weekly group yoga classes involving an expected 8 to 12 other women as well as practice study-specific yoga techniques at home for at least one hour per week for a total of 12 weeks. Women randomized to the muscle stretching and strengthening (a.k.a physical conditioning) control group will take part in twice weekly group physical conditioning classes and practice physical conditioning exercises at least one hour per week at home for 12 weeks. During the 12- and 24-week post-intervention follow-up period, participants will also be encouraged to continue practicing yoga or stretching/strengthening exercises for at least two hours per week.

To address objective 1, we will use validated voiding diaries to examine changes in frequency of total incontinence during the 12-week intervention, as well as explore persistence of effects on incontinence frequency after the end of the intervention programs. To address objective 2, we will further examine changes in frequency of different clinical types of incontinence as well as daytime and nighttime incontinence during the 12-week intervention, as well as the post-intervention follow-up periods. To address objective 3, we will analyze data from validated questionnaires in order to examine changes in incontinence-related functioning and quality of life over these same time periods. To address objective 4, we will assess changes in pelvic floor strength assessed during pelvic exam, autonomic

function assessed by heart rate variability and impedance cardiography measures, and physical function assessed by questionnaire as well as physical performance testing.

This study design is designed to provide rigorous evidence to evaluate the efficacy of the yoga intervention for treatment of incontinence in women, as well as exploring multiple potential mediators of improvement in incontinence and incontinence-related quality of life.

4. SELECTION AND ENROLLMENT OF PARTICIPANTS

Eligibility criteria have designed to identify a study population that is generalizable to most ambulatory middle-aged and older women with incontinence who do not have complicated urologic histories and also do not have a major contraindication to practicing yoga.

4.1 Inclusion Criteria

Candidates must meet all of the following criteria to participate in the study:

- Women aged 45 years or older who report urinary incontinence starting at least 3 months prior to screening
- Self-report at least 3 urinary incontinence episodes on a screening 3-day voiding diary (i.e., an average of at least one episode per day)
- Self-report urgency-predominant incontinence (i.e., at least half of incontinence episodes being urgency-type), stress-predominant incontinence (i.e., at least half of episodes being stress-type), or mixed-type incontinence (i.e., an equal number of stress- and urgency-type episodes) on the screening voiding diary
- Willing to refrain from initiating medical treatments that may affect their incontinence or voiding pattern during the study intervention period

4.2 Exclusion Criteria

Candidates meeting any of the following criteria at baseline will be excluded from participation:

- Participation in at least weekly formal or organized yoga classes or instruction within the past 3 months; or any prior yoga therapy directed specifically at improving urinary incontinence or pelvic floor dysfunction
- Participation in at least weekly organized physical conditioning classes or instruction in the past 3 months (i.e., classes involving muscle stretching and strengthening exercises, not including aerobic classes that do not emphasize muscle stretching or strengthening).
- Currently pregnant (by self-report or screening urine pregnancy test), gave birth within the past 6 months, or planning pregnancy during the study period (approximately 9 months)
- Current urinary tract infection (screening dipstick urinalysis with leukocyte estrace, nitrites or blood) or a history or 3 or more urinary tract infections in the preceding year
- Report history of neurologic conditions such as stroke, multiple sclerosis, spinal cord injury, or Parkinson’s disease, or a lumbosacral spine condition associated with neurological symptoms
- Unable to walk up a flight of stairs or at least 2 blocks on level ground without assistance (i.e., functional capacity < 4 METs)
- Unable to get up from a supine to a standing position in 10 seconds or less and without assistance

- Morbid obesity defined by a measured body mass index of >40 kg/m^2 at the screening evaluation.
- Report any history of prior anti-incontinence or urethral surgery (not including urethral dilation), pelvic cancer, or pelvic irradiation for any reason
- Report use of bladder botox, electrostimulation, bladder training, or pelvic floor exercise training (with certified practitioners) in the past 3 months
- Report other surgery to the pelvis (hysterectomy, oophorectomy, vaginal surgery, bladder surgery, colon surgery) within the past 3 months
- Report use of medications with the potential to affect incontinence (anticholinergic bladder medications, tricyclic antidepressants, mirabegron, loop diuretics) within the past month
- Report starting stopping, or changing the dose of a medication with the potential to affect depression or anxiety symptoms (i.e., selective serotonin reuptake inhibitors, anxiolytics/sedatives, antipsychotics) within the past 1 month, or plans to start, stop, or change to dose of such a medication during the study intervention period
- Report use of medical devices (i.e. pessary) for incontinence within the previous month (participants may stop use of device and re-present for study)
- Report history of interstitial cystitis, fistula or hole in bladder or rectum, or birth defect leading to urine leakage
- Report symptomatic pelvic organ prolapse (assessed using a standardized question, "Have your pelvic organs (uterus, bladder, or rectum) been dropping out of your vagina causing a feeling of bulging, pressure, or protrusion or a sensation like your "insides are coming out"?")
- Report history of vulvodynia, chronic pelvic pain, or pain when practicing pelvic floor exercises
- Report conditions that, in the judgment of the investigators, render potential participants unlikely to follow the protocol, including plans to move, substance abuse, significant psychiatric problems, or dementia
- Participation in another research study that involves investigational drugs or devices that could potentially confound the results of this study
- Unable to understand study procedures, complete study interviews, or and provide informed consent in English

4.3 Study Enrollment Procedures

Recruitment: Participants will be recruited from multiple locations in northern California using a multi-component approach that has been employed successfully in past clinical trials led by the investigators. This includes community-based media efforts (newspaper and radio advertisements, mass community mailings, notices posted in community and senior centers), recruitment from a database of women who have given permission to be contacted for women's health research studies based at the University of California San Francisco (UCSF) or Stanford University, recruitment of current and/or former female patients at UCSF or Stanford University identified through searches of electronic clinical databases, and posting or display of recruitment fliers in clinician offices (such as general internal medicine, geriatrics, gynecology, or alternative medicine). Recruitment will be organized in waves to assemble successive cohorts of women who are confirmed to meet all eligibility criteria and who are available to attend upcoming group yoga or muscle stretching/strengthening classes.

Screening: Preliminary eligibility will be determined by research coordinators through a Screening Telephone Interview followed by an in-person Screening Clinic Visit. During the Screening Telephone Interview, coordinators will follow a standardized screening interview script to assess preliminary eligibility related to age, gender, incontinence history, and exclusionary conditions and medications, as well as document reasons for ineligibility on the screening interview form. Candidates who appear preliminarily eligible at the end of the telephone interview will be scheduled to attend an in-person Screening Clinic Visit. After providing written informed consent at this visit, women will undergo more detailed assessment of incontinence symptoms, use of clinical incontinence therapies, exclusionary medical conditions, exclusionary medications, urine dipstick and pregnancy testing (for women of childbearing potential), and brief physical mobility assessment, with reasons for eligibility or ineligibility documented on the screening clinic intake form.

At the end of the Screening Clinic Visit, women who appear eligible will be given a blank voiding diary and instructions for completing it at home over a 3-day period and returning it to study staff at a Baseline Clinic Visit. Those who are confirmed to have stress-predominant, urgency-predominant, or mixed urgency-stress incontinence occurring at least 3 times over the 3-day diary period and meet all other inclusion/exclusion criteria at the Baseline Clinic Visit will be considered eligible for randomization to one of the two intervention groups.

Randomization: During each recruitment/randomization wave, eligible women will be randomized in a 1:1 ratio to one of the two intervention groups. Randomization will be stratified by clinical type of incontinence (i.e., stress, urgency, or mixed), to ensure adequacy of randomization within these three types. Randomization will be implemented by computer algorithm using randomly permuted blocks of sizes 2 and 4, with proof of eligibility required before assignment. To avoid manipulation, standard allocation concealment procedures will be followed (see subsequent section on Randomization for more detail).

To avoid excessive lag time between randomization and the start of the therapeutic yoga or stretching/strengthening control programs, randomization will take place no earlier than two weeks before the intervention program orientation sessions. For candidates whose Baseline Clinic Visit falls within this two-week window, randomization will take place directly at the Baseline Clinic Visit. For candidates who complete their Baseline Clinic Visit more than two weeks before the start of the yoga or control intervention programs, a Randomization Telephone Call will be scheduled to complete randomization within the two-week window.

5. STUDY INTERVENTIONS

5.1 Yoga Program

Overview and rationale: The 12-week yoga program will provide instruction and practice in a variety of yoga postures and techniques that have been selected for their potential to improve bladder control as well as their safety and feasibility for the target population. The study yoga program will be based primarily on Iyengar yoga, a form of Hatha yoga that is known for its potential therapeutic applications, has been employed successfully in other studies of yoga for different indications,[36-44] and differs from other Hatha yoga styles (power yoga, bikram yoga) in multiple ways that are likely to maximize both efficacy and safety for this study. Important features of

Iyengar/alignment-based yoga include: 1) emphasis on precise anatomical and postural alignment during practice of postures; 2) incorporation of props to minimize risk of injury and accommodate those with lower strength or flexibility; 3) emphasis on mindful awareness during practice of postures rather than rapid cycling through postures.

The study yoga program, which was pre-tested and refined in the investigators' prior pilot studies, is designed to maximize women's awareness of and control over the pelvic floor and improve underlying autonomic balance and physical conditioning, while still being feasible for ambulatory women across a wide range of ages, as well as grounded in techniques common to Iyengar yoga at large.

Content and postures: The yoga program will focus on a core set of 16 postures that are widely used in yoga practice and can be safely adapted for ambulatory women across a range of ages, including those with mildly or moderately decreased flexibility or mobility. These include both active postures engaging the pelvic floor and more passive postures promoting relaxation: Baddha Konasana (bounded angle pose), Malasana (squat pose), Bharadvajasana (seated twist pose), Supta Baddha Konasana (reclined cobbler's pose), Parsvottasana (intense side stretch pose), Salabhasana (locust pose), Savasana (corpse pose), Salamba Set Bandhasana (supported bridge pose), Parsvokonasana (side angle pose), Utkatasana (chair pose), Trikonasana (triangle pose), Vajrasana (thunderbolt), Virabhadrasana 2 (warrior 2 pose), Tadasana (mountain pose), Supta Padagushthasana (reclining big toe pose), and Viparita Karani Variation (legs up the wall pose).

Staff qualifications and training: Each yoga class series will be led by a primary instructor who must: 1) have at least 2 years of experience teaching yoga in the community; 2) review a detailed, study-specific written instructor manual describing all of the postures and yoga techniques to be used in the study program; 3) complete at least 2 hours of in-person training in study-specific yoga techniques with one of the study yoga expert consultants; 4) complete additional study-specific training in clinical trial procedures, documentation of participant attendance, review of home practice logs, and recording of yoga postures practiced during group classes on appropriate study forms; 5) carry and submit proof of personal liability insurance (minimum of $2 million coverage); and 6) confirm his or her willingness to adhere to the study intervention procedures. For each class series, a back-up instructor will also be identified who can teach a class in the event that the primary instructor is ill or is unable to teach due to an emergency.

Orientation and classes: Prior to group instruction, participants in each class series will attend a 90-minute group orientation led by the appointed class instructor. This will include an overview of the general principles of Iyengar/alignment yoga, an orientation to the structures of the pelvis, spine, and lower extremity, and an introduction to the postures to be practiced and the props to be used in the study. After the orientation, women will attend 90-minute group classes twice weekly for 12 weeks, led by the same instructor. For classes with 10 or more students, the instructor may be supported by an assistant who will help to provide personalized attention to students. During group classes with no more than 15 participants, the instructor will guide women in practicing yoga postures, calling attention to ways in which postures can improve pelvic floor function and adapt postures to accommodate physical limitations. Women will also be taught to avoid habits that may worsen incontinence during practice, such as squeezing of abdominal rather than pelvic muscles. Following a study-specific guide, instructors will gradually

introduce postures as classes progress, with the goal of making participants comfortable with all core postures by the end of 6 weeks and fully confident in performing postures by 12 weeks. During the COVID-19 pandemic, class instruction may be converted to remote platforms (i.e., may take the form of interactive on-line video-based classes rather than in-person classes).

Home practice: In addition to attending group classes, participants will be asked to practice yoga at home at least one additional hour per week and will be given a diary to keep track of the dates and times of practice. Women will also be given a written manual, pre-tested in the investigators' pilot studies, that includes pictures and descriptions of each yoga posture to guide them in home practice. Each participant will be given a limited set of yoga props (a mat, belt, and 2 blocks) for home practice. Women will be encouraged to practice with other members of their classes, but will be discouraged from practicing with individuals who are not in their class and from taking part in non-study yoga activities that might dilute study techniques.

Quality and safety: The study program will incorporate multiple modifications to help women across the age range to perform postures in ways that minimize risk of muscle strain or injury. Participant manuals will include specific instructions on adapting each posture and/or using props to accommodate problems with mobility, flexibility, or balance. To promote quality and safety, the instructors leading classes will be required to have at least 2 years of experience teaching yoga and have completed dedicated, study-specific training with the study's expert consultants. Following procedures established in the pilot study, a consultant will visit at least one class in each series to ensure that postures are being taught according to protocol, evaluate students' success in learning yoga, and ensure fidelity across instructors. Twice monthly conference calls will be conducted to help instructors trouble-shoot issues that arise in instruction.

Post-intervention program continuation: After the end of the main 12-week intervention period, participants will be encouraged to continue to practice yoga at least two hours per week at home, although group intervention classes will no longer be offered through the study.

5.2 Muscle Stretching/Strengthening (i.e., Physical Conditioning) Control Program

Overview and rationale: This trial will include a 12-week non-specific muscle stretching/strengthening program that will provide a rigorous time-and-attention control for the yoga program, but has been designed to avoid engaging the pelvic floor or promoting mindful relaxation. To minimize differential expectations of treatment success, women will be told that they are enrolling in a study of two different types of low-impact physical activity-based interventions (yoga versus physical conditioning) to enhance management of incontinence. Control group exercises have been adapted from exercises used in prior studies conducted by the investigators, such as the PRYSMS trial of restorative yoga vs. stretching for metabolic syndrome (designed by Sarah Pawlowsky, DPT, consultant on this proposal), and pilot-tested in the investigators' prior pilot study of yoga versus physical conditioning for women with incontinence. Similar to postures in the yoga program, the exercises in the stretching/strengthening program have been selected for their potential to be performed safely by women across a range of ages and flexibility levels.

Content and exercises: After a brief education about sitting and standing posture,

the control intervention program will focus on a core set of muscle stretching and strengthening exercises that will be performed sitting in a chair: levator scapulae, upper trapezius, scalenes, shoulder horizontal adduction, triceps, pectoral stretch with hands behind head, wrist flexors and extensors, trunk flexion, trunk extension, trunk side-bend, trunk rotation, and hamstring stretches. Additional chair exercises will incorporate a stretching strap: pectoral stretch with strap, gastrocnemius stretch with strap, and hand behind back with strap. A few stretches will be performed on an exercise mat on the floor: supine single knee to chest, supine hamstrings stretch with strap, quadriceps stretch with strap, supine shoulder flexion with stretch strap, and seated adductor stretch. Several exercises will be performed in the standing position: gastrocnemius, soleus, shoulder flexion, and shoulder abduction stretches.

Staff qualifications and training: Each stretching/strengthening class series will be led by a primary instructor who: 1) is either certified as a personal trainer through the National Academy of Sports Medicine, National Strength and Conditioning Association, or the American College of Sports Medicine, or National Strength and Conditioning Association (NSCA), or licensed as a physical therapist by the Physical Therapy Board of California; 2) has at least 2 years of experience working as a physical therapist or physical trainer in either individual or group settings; 3) will have reviewed a detailed, study-specific training manual describing all of the stretching/strengthening exercises to be used in the study program; 4) will have completed at least 2 hours of in-person training in study-specific techniques with study expert consultant; 5) will have completed additional study-specific training in clinical trial procedures, documentation of participant attendance, review of home practice logs (for adherence), and recording of exercises performed during group classes on appropriate study forms; 6) carries and submits proof of personal liability insurance (minimum of $2 million coverage); 7) confirms his or her willingness to adhere to the study intervention procedures. For each class series, a back-up instructor will also be identified who can teach a class in the event that the primary instructor is ill or is unable to teach due to an emergency.

Group orientation and classes: Similar to the yoga program, the stretching/strengthening control program will begin with a group orientation led by a physical therapist/trainer, who will provide an overview of the stretching/strengthening exercises to be covered in the program. This will be followed by 90-minute twice weekly group classes, each with no more than 15 participants, led by the same therapist/trainer, who will have received in-person study-specific training from a study expert physical therapist consultant. Classes will be designed to make women comfortable with all exercises by 6 weeks and fully confident in performing exercises by 12 weeks. During the COVID-19 pandemic, classes may be coverted to remote platforms (i.e., may take the form of interactive on-line video-based classes rather than in-person classes).

Home practice: In addition to attending group classes, women will be asked to perform stretching/strengthening exercises at home at least one additional hour per week and record the dates and times of practice in a log. Women will receive a detailed manual with pictures and descriptions of each stretching/strengthening exercise to guide them in home practice, as well as a stretch strap and exercise mat to use at home.

Quality and safety: Instructors leading physical conditioning classes will be physical therapists or trainers who have at least 2 years of experience working with adults of a range of ages and have completed dedicated in-person training with Sarah

Pawlowsky, DPT. Dr. Pawlowsky will provide quality monitoring and assess women's success in learning stretching/strengthening exercises by making an in-person visit to at least one class in each series. She will also lead twice monthly conference calls with instructors, similar to quality monitoring for the yoga program.

Post-intervention program continuation: After the end of the main 12-week intervention period, participants will be encouraged to continue practice stretching/strengthening exercises at least two hours per week at home, although group intervention classes will no longer be offered through the study.

5.3 Concomitant Interventions

5.3.1 Required Interventions

Participants in both intervention groups will also receive a pamphlet at the Baseline Clinic Visit that provides basic patient-directed information about first-line behavioral self-management of incontinence, including pelvic muscle exercises, timed urination, and urge suppression. This pamphlet is consistent with usual care of incontinence in the general community, and has been used in previous studies of experimental treatments for incontinence conducted by the investigators.[45] Participants will be queried about use of these techniques at baseline, at the 6-week and 12-week clinic visits, and as part of the 12- and 24-week post-intervention assessment, so that information about co-interventions can be incorporated into data analyses if appropriate.

Since information about behavioral self-management of incontinence is available from multiple websites and public resources, systematic provision of this information will help avoid differential use of self-management techniques between treatment groups, and will also reflect expected concomitant use of these techniques with yoga in clinical practice. However, participants will be discouraged from pursuing more formal training or coaching in behavioral incontinence treatment strategies from pelvic floor physical therapists or other specialized healthcare practitioners during the trial intervention period (see prohibited interventions below).

5.3.2 Prohibited or Discouraged Interventions

Participants will be asked to refrain from using other standard clinical treatments for incontinence during the 12-week intervention period. These include medications with the potential to affect incontinence (anticholinergic bladder medications, tricyclic antidepressants, mirabegron), invasive or surgical bladder treatments, nerve stimulation therapy, medical devices used to improve bladder symptoms (e.g., pessary), and behavioral treatment programs administered by certified practitioners (e.g., pelvic floor rehabilitation therapy, biofeedback programs).

Participants will be queried about use of these treatments at baseline, at the 6-week and 12-week clinic visits. If participants are found to be using these treatments during the 12-week study intervention period, they will not be required to terminate the study early, but use of outside treatment will be recorded as a protocol deviation, and information about use of these additional treatments may be taken into account in per protocol data analyses.

During the during the 12- and 24-week post-intervention follow-up periods, participants will also be encouraged to avoid using other clinical treatments for incontinence, and instead focus on continued home practice of study yoga or muscle stretching/strengthening exercises to manage their symptoms. Participants will be queried about use of these treatments at the 12- and 24-week follow-up assessments, so that information about use of these treatments can be incorporated into data analyses. However, use of other clinical incontinence treatments during the 12- and 24-week post-intervention follow-up periods will not be considered a protocol deviation.

Additionally, participants will be asked to refrain from starting or changing the dosage of medications that could affect depression or anxiety symptoms (e.g., antidepressants, sedatives/hypnotics, or antipsychotic agents) during the 12-week intervention period or 12- or 24-week post-intervention follow-up period, although maintenance of stable dosages of these medications will be acceptable.

5.4 Adherence Assessment

Adherence to group yoga and physical conditioning classes will be documented by the study class instructors using standardized attendance logs. Attendance logs will be returned to clinical coordinators on a weekly basis throughout the 12-week yoga and control intervention programs. Clinical coordinators will immediately contact any participant who misses a scheduled class to ask about reasons for missing the class, reinforce the importance of class attendance, and troubleshoot any barriers to attendance.

Adherence to home practice of yoga or stretching/strengthening exercises will be tracked using home practice logs. Participants will be instructed to record the date, time, and duration of each home practice session in their logs, as well as the specific yoga poses or stretching/strengthening exercises practiced during each session. Home practice logs will be returned to the clinical coordinators on a weekly basis during the 12-week intervention programs. Clinical coordinators will contact any participant whose log indicates that she is not practicing yoga or stretching/strengthening exercises at least one hour a week at home. Additionally, home practice logs will be returned 12 and 24 weeks after the end of the intervention program to assess home practice during the post-intervention follow-up period.

Every effort will be made to encourage participants to complete all intervention classes and practice sessions. Prior to randomization, study coordinators will stress the importance of adherence to group classes and home practice, and only those women who indicate that they are available on scheduled class dates and willing to practice yoga or stretching/strengthening exercises at home as recommended will be eligible for randomization. The importance of adherence will again be stressed at the orientation sessions for each intervention class series, as well as during follow-up telephone calls at 2 weeks and a follow-up visit at 6 weeks. Additionally, attendance sheets from yoga and stretching/strengthening classes will be carefully monitored, and home practice logs will be reviewed weekly, so that study staff can immediately call any women who miss classes or fail to document home practice in order to reinforce the importance of attendance/practice and troubleshoot barriers to adherence.

6. STUDY PROCEDURES

6.1 Study Measures

6.1.1 Urinary Incontinence

The primary efficacy outcome, frequency of any urinary incontinence, will be assessed using a 3-day voiding diary that has been shown to be a valid and reliable method for documenting change in incontinence symptoms and is widely used in clinical trials of incontinence treatments.[46,47] At screening/baseline, 6 weeks, 12 weeks, and 12- and 24- weeks after the end of the yoga and control intervention programs (i.e., 24 and 36 weeks), each participant will receive written instructions and a blank diary to take home, and will use the diary to record all voiding and incontinence episodes over a 3-day period and classify incontinence episodes by clinical type (urgency, stress, other). Upon return of the diary, an analyst will abstract data to determine the number of total as well as urgency and stress incontinence episodes per day. For diaries that are completed after randomization, data will be abstracted by an analyst who is blinded to treatment assignment.

6.1.2 Incontinence-Related Quality of Life

To address incontinence-related quality of life, participants will complete the following validated questionnaire measures at baseline, 6 weeks, and 12 weeks, as well as 12 and 24 weeks after the end of the yoga and stretching/strengthening control programs:

A. Incontinence Impact Questionnaire (IIQ)-- a validated, 28-item measure of the impact of incontinence on 4 domains of functioning and quality of life (physical activity, emotional health, relationships, and travel).[48,49] Scores range from 0 to 100; higher scores indicate worse impact.
B. Urogenital Distress Inventory-6 (UDI-6)-- a validated, 6-item measure that assesses subjective distress from frequent urination, urgency incontinence, stress incontinence, small-volume leakage, difficulty emptying the bladder, and genital pain.[49] Scores are scaled from 0 to 100.
C. Patient Perception of Bladder Condition (PPBC)-- a validated single-item measure assessing the degree to which respondents consider their condition to be a problem on a 6-point scale.[50,51]
D. Pelvic Organ Prolapse/Incontinence Sexual Questionnaire, IUGA-Revised (PISQ-IR)- a validated 20-item measure of sexual function in women with pelvic floor disorders (PFDs), designed to be appropriate for both sexually active and inactive women.[52]

6.1.3 Depression, Anxiety, and Perceived Stress

To evaluate depression, anxiety, and perceived stress symptoms that may be associated with incontinence, participants will complete the following validated questionnaire measures at screening/baseline, 6 weeks, and 12 weeks, as well as 12 and 24 weeks after the end of the yoga and control intervention programs:

A. Center for Epidemiologic Studies Depression Scale (CES-D): Depressive symptoms will be assessed by the CES-D, a 20-item measure that has been widely used in clinical trials, including bladder interventions, and is sensitive to change.[53] Total scores range from 0 to 60.

B. Spielberger State Trait Anxiety Inventory (STAI): Somatic anxiety (i.e., the affective component of anxiety believed to be related to autonomic physiological arousal response) will be measured using the trait component of the STAI, a 20-item self-administered measure validated in clinical populations, including patients with bladder symptoms, with scores ranging form 20 to 80.[54,55]
C. Hospital Anxiety and Depression Scale (HADS): Cognitive anxiety (i.e., the mental component of anxiety associated with fear of failure) will be measured by the HADS, a validated self-administered questionnaire that includes a 7-item Anxiety Subscale[56] shown to be sensitive to change in incontinence trials.[57] Scores range from 0 to 21, with higher scores indicating greater anxiety.
D. Perceived Stress Scale (PSS): Perceived stress will be assessed by the PSS, a 10-item measure of thoughts and feelings related to perceived stress in the past month, validated in a probability sample of the United States.[58] Scores range from 0 to 40; higher scores indicated greater stress.

6.1.4 Yoga/Physical Conditioning Self-efficacy and Competency

A. Yoga Posture Self-Efficacy: After randomization, 6 weeks, and 12 weeks, women in the yoga group will complete a structured questionnaire (modeled after an existing self-efficacy measure [59] and pre-tested in the investigators' previous pilot study) to indicate how confident they are that they can perform each of the postures featured in the program (from 'not at all' to 'extremely confident'). A corresponding questionnaire will be administered to the muscle stretching/strengthening control group participants.
B. Independent Yoga Competency Assessment: To provide a more objective assessment of women's success in learning yoga, a yoga expert consultant will attend one class at the end of each series and observe women as they practice yoga. The consultant will rate each woman's success in performing each yoga posture on a 5-point scale, ranging from "not at all" to "extremely" successful. In the muscle stretching/strengthening control group, a study expert physical therapist consultant will provide similar assessments.
C. Yoga Practice Adherence Self-Efficacy: After randomization, 6 weeks and 12 weeks, as well 12 and 24 weeks after the end of the intervention programs, women will complete a measure of confidence in adhering to yoga or stretching practice, modeled after existing physical activity adherence self-efficacy scales [60] and tested in the pilot study. Women will indicate on a 5-point scale how confident they are that they can practice yoga or stretching when they: 1) are tired, 2) are in a bad mood, 3) have limited time, 4) are away from home, and 5) are not regularly attending yoga classes. Scores will range from 0 to 20.
D. Credibility and Expectations of Treatment: To assess whether women perceive the yoga and physical conditioning interventions as equally credible and have similar expectations for improvement in incontinence, we will administer a modified version of an expectancy/credibility measure used in other women's health studies at baseline.[61]

6.1.5 Physical Function/Performance Measures

To guide exploration of changes in physical function/conditioning as a potential mediator of intervention effects in women with incontinence, participants will complete the following assessments at baseline, 6 weeks, and 12 weeks:

A. PROMIS Adult Physical Function Profile short-form: This self-administered questionnaire measure of perceived physical function assesses functioning of the upper extremities, lower extremities, and central regions (neck, back), and has previously been validated across age and race-ethnicity groups.[62]
B. Balance testing: Participants will be asked to undergo successive semi-tandem, tandem, and one-legged balance stand tests, in which their ability to hold each position for up to 30 seconds will be assessed.[63,64]
C. 30-second chair stand: Participants will be asked to stand up from a standard chair to a fully extended standing position as many times as possible within 30 seconds with their arms folded across their chest. The number of completed repetitions achieved in 30 seconds will be recorded, with higher number indicating more strength or power.[65]
D. 2-minute step test: In this test of aerobic endurance for older adults,[69,70] participants subjects are asked to step in place as many times as possible in a 2 minute period, each time raising the knee to a level midway between the patella and iliac crest. Higher scores indicating greater fitness.

6.1.6 Cardiac Autonomic Function Measures

To guide exploration of changes in autonomic nervous system function as a potential mediator of intervention effects in women with incontinence, participants will undergo the following measures at baseline and 12 weeks. During the COVID-19 pandemic, participants may decline to undergo these measurements that require them to come into close proximity (less than 6 foot distance) with study personnel.

A. High frequency heart rate variability (a.k.a. respiratory sinus arrhythmia, or RSA) refers to the degree to which heart rate accelerates and decelerates during the respiratory cycle, and has been shown to be a reliable marker of cardiac parasympathetic activity.[71-73] Evaluation of rest-to-stress changes in RSA allows for assessment of abnormalities in adaptive vagal regulation, previously shown to be associated with bladder instability and urine leakage in response to stressful events.[20-22] Procedures for assessing RSA, monitoring data quality, and interpreting findings have been designed by co-investigator Wendy Mendes, PhD, who has authored one of the main textbooks on autonomic assessment,[74] published four methodological articles and chapters on this topic,[75-78] and collaborated with the research team on other NIH-funded studies.[79] Heart rate variability measurements will be obtained according to standard protocols from the North American Society of Pacing & Electrophysiology,[80] in which two ECG electrodes will be attached to each participant's right and left wrists using pre-gelled sensors while in the seated position. Following an initial 10-minute "resting" recording, a 10-minute "stress" recording will be obtained while the participant completes two stress tasks, including a visual attention and tracking task[81] shown to reliably induce vagal

withdrawal in subjects across the lifespan.[75,76] Analyses using Mindware software will examine both resting and rest-to-stress change in RSA.

B. Systolic pre-ejection period (i.e., PEP, the time period between the left ventricle contracting and the aortic valve opening) will be examined as an established measure of sympathetic activity that can be measured non-invasively using impedance cardiography.[72,73] For this study, PEP will be measured using the impedance cardiography module of the same Biopac MP100 system used to assess RSA above. According to established protocols[82] also used in other NIH-funded studies by the team,[79] a standard tetrapolar band electrode system will be attached to each participant while in the seated position (two inner electrodes placed at the xiphisternal joint and the base of the neck, outer electrodes placed 3 cm distally to the inner electrodes) and connected to the impedance cardiograph module. Impedance cardiogram recordings will be obtained over the same time interval as RSA recordings, including a resting period and a stress period. This will include a computer-based stress task designed by co-investigator Dr. Mendes to assess sympathetic reactivity,[83] which participants must complete with limited instruction and under time constraints. Analyses using Mindware software will examine both resting and rest-to-stress change in PEP.

6.1.7 Pelvic Floor Muscle Strength

To guide exploration of changes in pelvic floor muscle strength as a potential mediator of intervention effects in women with incontinence, participants will be invited to undergo the following optional assessment of pelvic floor muscle strength at baseline and 12 weeks. For this assessment, a clinically-trained assessor (doctor, nurse, or pelvic floor physical therapist) will perform a digital pelvic exam and ask the participant to squeeze her pelvic floor muscles during exam. The assessor will then rate pelvic muscle strength using the Brink scale, which includes 4-point scales in 3 categories—contraction pressure, displacement of the examiner's fingers, and duration of squeeze— to create an overall score of 0 to 12.[84-90] Since this is an optional assessment, participants who have a relative contraindication to pelvic exam or anticipate pain with pelvic exam can decline to undergo this measurement. During the COVID-19 pandemic, participants may also decline to undergo these measurements that require them to come into close proximity (less than 6 foot distance) with study personnel. For assessments performed after randomization, the assessor will be blinded to intervention assignment.

At the Stanford clinical site, participants will also be invited to undergo perineometer assessment of pelvic floor muscle strength by a board-certified urogynecologist at the same timepoints (baseline and 12 weeks). Participants will be asked to contract their pelvic floor muscles, and contraction strength will be measured with a Laborie Urostym digital perineometer. This device is currently used clinically and approved for pelvic floor physical therapy and biofeedback. Measurements obtained by perineometer have previously been shown to have good inter-rater and test-retest reliability. Measurements will include minimum, maximum, and average strength of each pelvic floor muscle contraction measured. Duration of contraction will also be recorded. A total of three contractions will be measured by digital perineometry. Measurements

will be recorded in cm H2O or mm Hg. Each patient will be assigned a vaginal sensor that will be processed according to manufacturer's recommendation and used for the baseline and follow-up exams. Since this is an optional assessment, participants who have a relative contraindication to pelvic exam or anticipate pain with pelvic exam can decline to undergo this measurement.

6.1.8 Additional Screening and Covariate Measures

The following data will be collected to assess eligibility, assess whether treatment groups are comparable at baseline, and guide statistical adjustments in the event groups are not balanced:

A. Demographic history including date of birth, gender/sex identity, race/ethnicity, education history, employment history, marital status (screening/baseline)
B. Urologic/urogynecologic history including age of onset of incontinence, past and current treatment of incontinence, history of pelvic surgery, history of pregnancy and delivery, menstrual/menopausal status, sexual activity status (screening/baseline)
C. General medical history including general health assessed by the CDC Healthy Days module,[91] selected medical conditions associated with incontinence such as diabetes, tobacco and alcohol use, and physical activity level (screening)
D. Current medication use (screening/baseline and 12 weeks)
E. Health-related habits such as tobacco/alcohol use (screening); fluid and caffeine intake using the fluid intake amount and fluid intake behavior sections of the Questionnaire-Based Voiding Diary[92-94] (baseline, 6 weeks, 12 weeks, and 12 and 24 weeks post-intervention); prior yoga experience, prior group-based physical activity instruction (screening), physical activity level by the International Physical Activity Questionnaire[95,96] (screening)
F. Sleep function assessed using the Pittsburgh Sleep Quality Index (PSQI),[97,98] a validated questionnaire evaluating sleep quality, latency, efficiency, and problems, and a the Pittsburgh Sleep Diary, a daily self-report measure of sleep duration and disruption used to enhance or complement data obtained from actigraphy in multiple past studies of older adults[99-101] (baseline, 6 weeks, and 12 weeks, as well as 12 and 24 weeks after the end of intervention programs).
G. Physical exam measures including measured height, weight, heart rate, and blood pressure (screening/baseline and 12 weeks)

6.2 Evaluations

6.2.1 Screening Evaluations

Screening evaluations will be conducted by study coordinators based at outpatient facilities affiliated with UCSF or Stanford University. Screening procedures will include a brief preliminary Screening Telephone Interview, followed by an in-person Screening Clinic Visit, completion of a 3-day voiding diary at home, and review of the diary at an in-person Baseline Clinic Visit. All screening procedures must be completed within a 90-day period in order for participants to be eligible for randomization. If screening procedures cannot be completed within 90 days, initial screening procedures must be repeated

to ensure that participant eligibility has not changed prior to randomization.

6.2.2 Consenting Procedure

Prior to administration of any data collection instruments, signed consent will be obtained by a research coordinator at the beginning of the Screening Clinic Visit. A single informed consent process will be used that will cover both the screening and post-randomization procedures. All participants must be able to read and understand the consent form in English and must provide written informed consent before enrolling in the study. Following the UCSF IRB-approved template, the consent form will describe the purpose of the study, the procedures involved in recruiting, randomized, and monitoring participants, and the potential risks and benefits associated with participation.

A study coordinator will first explain the study procedures to the potential participant, referring as necessary to the detailed IRB-approved consent form. The coordinator will then give a copy of the consent form to the potential participant to read. After the potential participant has read the consent form, she will be asked if she has any questions or concerns about the study. Once these questions/concerns have been addressed, the participant will be asked to sign the consent form, and will be given a copy of the consent form to take home for future reference.

Pursuant to California Health & Safety Code 24172, each participant will also receive a copy of the Experimental Subject's Bill of Rights.

6.2.3 Screening Procedures

Telephone Screening Interview

- Women who call in response to recruitment advertisements or who have previously given permission to be contacted about opportunities to participate in women’s health research at UCSF will be provided with a brief overview of the study goals and procedures by a clinical coordinator over the telephone.
- If interested, candidates will complete a brief telephone survey to assess preliminary eligibility (including age, gender, duration/frequency/type of incontinence, current and past incontinence treatment, prior yoga and muscle strengthening exercise practice, physical mobility, exclusionary conditions and medications, availability for upcoming group intervention classes).

Screening Clinic Visit

- At this visit, a clinical coordinator will explain the requirements of the study while referring to the detailed informed consent form, and candidates will provide written informed consent if they are still interested in proceeding with the study.
- Candidates will complete questionnaires about their demographic, urologic/urogynecologic, and medical history to determine if they meet criteria related to age, gender, duration/frequency/type of incontinence, current and past incontinence treatment, prior yoga and muscle strengthening exercise practice, exclusionary conditions, and medications
- Over-the-counter and prescription medications will be reviewed to determine if candidates are taking any exclusionary medications.

- Height, weight, and resting blood pressure and pulse measurements will be obtained.
- Basic physical mobility including ability to rise from a supine to standing position will be assessed.
- A clean-catch urine sample will be collected to assess for possible urinary tract infection and rule out pregnancy (for candidates who are still of child-bearing potential).
- Potentially eligible women will be instructed on completing a 3-day voiding diary to document all incontinence and voiding episodes at home.
- A Baseline Clinic Visit will be scheduled at least 3 days later for review of the completed voiding diary and determination of final eligibility.

<u>Baseline Clinic Visit</u>

- Candidates will return with the completed voiding diary, and results will be reviewed by a clinical coordinator. Candidates whose diaries indicate predominantly urgency, stress, or mixed-type incontinence, document at least 3 incontinence episodes per 3-day diary, and meet all other eligibility criteria will be eligible to continue.
- Women will complete structured questionnaires about depression and anxiety, incontinence-specific quality of life, and sleep quality.
- Physical function will be assessed by questionnaire as well as direct physical performance testing.
- Cardiac autonomic function will be assessed through heart rate variability and impedance cardiography measurements.
- Participants will be invited to undergo pelvic floor muscle strength assessments involving a digital pelvic exam (and/or perineometer assessment if the visit is at the Stanford site). The assessment may be performed directly during the Baseline Visit, or an alternate clinic assessment may be scheduled specifically for this exam.
- Participants will be given a brief written pamphlet about usual first-line behavioral management of incontinence (see description in interventions section above).
- If the Baseline Clinic Visit takes place within 2 weeks of the start of the first intervention program session, eligible participants will be randomized to one of the two interventions at this visit.
- If the Baseline Clinic Visit takes place more than 2 weeks before the first intervention program session, eligible participants will be scheduled for a Randomization Telephone Call within two weeks of the start of the program to determine their intervention assignment.

6.2.4 Randomization and Blinding

<u>Randomization</u>

Women who are confirmed to be eligible and available to take part in upcoming group intervention class series will be randomized in a 1:1 ratio to one of the two intervention groups. Randomization will ideally take place within 1 month of completion of screening procedures, and in no cases will be able to take place more than 2 months after completion of screening procedures. Randomization must also take place within 2 weeks of the start of the relevant group intervention class series. If the Baseline Clinic Visit occurs within 2 weeks of the start of the intervention program, eligible

participants will be randomized to one of the two interventions directly at this visit. If the Baseline Clinic Visit occurs more than 2 weeks before the start of the intervention program, eligible participants will be scheduled for a Randomization Telephone Call within two weeks of the start of the program to determine their intervention assignment.

During the Baseline Clinic visit or during the Randomization Telephone Call (whichever is appropriate), randomization will be performed according to a pre-established computer-algorithm using randomly permuted blocks of sizes 2, and 4. Randomization will be stratified by clinical type of incontinence (i.e., stress-predominant, urgency-predominant) to ensure adequacy of randomization among women with each major incontinence type. Prior to randomization, the research coordinator will complete a randomization checklist confirming that the participant meets all eligibility criteria. Additionally, participants' availability and willingness to attend upcoming yoga or control group classes will be re-confirmed before randomization assignment is obtained.

Standard allocation concealment procedures will be followed to avoid manipulation of randomization.[102] Prior to the start of recruitment, the randomization scheme will be developed by a statistical programmer who will have no contact with participants or role in data entry, cleaning, or analysis. To execute the randomization scheme, a research assistant who will also have no contact with participants or access to study data will then prepare a series of sealed, opaque envelopes containing group assignment, numbered consecutively with the randomization sequence numbers. When the participant is ready for randomization, study coordinators will enter the date, participant name, and study ID in the randomization log and select the next numbered envelope corresponding to the participants' type of incontinence to determine group assignment. Once a participant has been randomized, she cannot be re-assigned to a different intervention group. All randomization envelopes will be retained for review; randomization dates and times should follow the order of the sequence numbers, providing a check on validity. After randomization, participants will complete self-efficacy questionnaires specific to their assigned intervention (yoga or stretching/strengthening).

Blinding

Although participants cannot be blinded to intervention assignment (due to the behavioral nature of the interventions), study procedures have been carefully designed to avoid bias associated with knowledge of group assignment. First, to minimize differential expectations of treatment success, participants will be told that they are participating in a study of two different types of low-impact group activity-based interventions to enhance self-management of incontinence, and that we do not know which is more effective (which is true). The primary outcome will be assessed by voiding diaries, a measure that is resistant to reporting bias, and data from returned diaries will be abstracted by blinded analysts. Similarly, data from participant follow-up questionnaires assessing other symptom and quality-of-life outcomes associated with incontinence will be abstracted by blinded analysts.

Only study personnel who are involved in delivering the therapeutic interventions, assessing and promoting adherence to these interventions,

and/or monitoring adverse events related to these interventions will be aware of group assignment. All investigators, study analysts involved in abstracting efficacy outcomes data, and statistical programmers involved in ongoing data cleaning will be blinded to treatment allocation. Unless stipulated by the Data and Safety Monitor or required to assist with the clinical management of a participant experiencing a serious adverse event, these investigators and staff will remain blinded until after all participants have completed the 12-week clinic visit, trial data from the 12-week visit are edited and cleaned, and the trial dataset for the 12-week intervention period is locked.

6.2.5 Follow-up/Final Evaluations

Follow-up evaluations will consist of a 2-week telephone call, 6-week clinic visit, 9-week telephone call, 12-week clinic visit, and 24-week mail-in assessment:

2-Week Telephone Call

- Two weeks after the start of the yoga or control program, coordinators will call women to assess adverse events, address any concerns, and reinforce adherence to group classes and home practice of yoga or stretching/strengthening exercises.
- Adherence to and challenges in performing usual behavioral self-management strategies for incontinence will also be assessed.
- Women will be reminded to continue recording home yoga or stretching/strengthening practice in their practice logs and return their logs each week to the yoga class instructors. They will also be reminded to start filling out a second voiding diary 3 days before the 6-Week Clinic Visit.

6-Week Clinic Visit

- Six weeks after the start of the yoga or control program, women will return for a follow-up visit, bringing their second completed voiding diary.
- Voiding diaries will be retrieved by the research coordinator and delivered to a blinded analyst for data abstraction.
- Adverse events will be recorded using standardized forms (see safety monitoring section).
- Adherence to yoga or stretching/strengthening practice and incontinence self-management strategies will be re-assessed.
- Yoga and stretching/strengthening self-efficacy questionnaire measures will be administered.
- Participants will re-self-administer questionnaires about incontinence-related quality of life, depression and anxiety symptoms, and sleep quality.
- Participants will be asked about their use of other strategies to manage their incontinence.
- Physical function will be assessed by questionnaire as well as direct physical performance testing.
- Current use of prescription and over-the-counter medications will be re-reviewed.
- Women will be given a third voiding diary to complete before the 12-week visit, and the 12-week visit will be scheduled.

9-Week Telephone Call

- Nine weeks after the start of the yoga or control program, coordinators will call women to assess adverse events, address any concerns, and reinforce adherence to group classes and home practice of yoga or stretching/strengthening exercises.
- Adherence to and challenges in performing usual behavioral self-management strategies for incontinence will also be assessed.
- Women will be reminded to continue recording home yoga or stretching/strengthening practice in their practice logs and return their logs each week to the yoga class instructors. They will also be reminded to start filling out a third voiding diary 3 days before the 12-Week Clinic Visit.

12-Week Clinic Visit

- Twelve weeks after the start of the yoga or control program, women will return for another clinic visit, bringing their third completed voiding diary.
- Voiding diaries will be retrieved by the research coordinator and delivered to a blinded analyst for data abstraction.
- Adverse events will be recorded using standardized forms (see safety monitoring section).
- Yoga and stretching/strengthening self-efficacy questionnaire measures will be re-administered.
- Participants will re-self-administer questionnaires about incontinence-related quality of life, depression and anxiety symptoms, and sleep quality.
- Physical function will be re-assessed by questionnaire as well as by physical performance testing.
- Participants will be asked about their use of other strategies to manage their incontinence.
- Cardiac autonomic function will be assessed through heart rate variability and impedance cardiography measurements.
- Participants will be invited to undergo pelvic floor muscle strength assessments. The assessment may be performed directly during the 12-Week Clinic Visit, or an alternate clinic assessment may be scheduled specifically for this exam.
- Current use of prescription and over-the-counter medications will be re-reviewed.
- A satisfaction questionnaire will be administered to assess overall satisfaction with study procedures and change in incontinence symptoms.
- Women will be encouraged continue practicing yoga or stretching/strengthening exercises for at least two hours per week for the next 12 weeks and record this practice in their home practice logs.
- As an optional activity, selected participants in the yoga arm may be invited to take part in brief qualitative interviews to discuss their barriers and facilitators to learning and maintaining practice of yoga after this visit.

24-Week (a.k.a. 3-Month Post-Intervention) Assessment

- Approximately twelve weeks after completion of the intervention programs, coordinators will call participants to them to complete their fourth voiding diary at home (the blank diary will be mailed in advance to participants at home, and participants will return it using a pre-stamped and addressed envelope).
- Adverse events will be assessed by telephone, video, or in-person visit and recorded using standardized forms (see safety monitoring section).
- Women will also be asked to complete questionnaires addressing symptom, quality-of life, and sleep outcomes as well as yoga or stretching/strengthening practice adherence and self-efficacy at home (questionnaires will be mailed in advance to participants at home, or can be completed via an on-line survey).
- Participants will be asked about their continued practice of yoga or stretching/strengthening techniques and their use of other strategies to manage their incontinence.
- Participants will be asked to mail back home practice diaries documenting their continued practice of yoga or stretching/strengthening techniques.
- Women will be encouraged continue practicing yoga or stretching/strengthening exercises for at least two hours per week for the next 12 weeks and record this practice in their home practice logs.

<u>36-Week (a.k.a. 6-Month Post-Intervention) Assessment</u>

- Approximately 24 weeks after completion of the yoga or control program, coordinators will call participants to them to complete their fifth voiding diary at home (the blank diary will be mailed in advance to participants at home).
- Adverse events will be assessed by telephone, video, or in-person visit and recorded using standardized forms (see safety monitoring section).
- Women will also be asked to complete questionnaires addressing symptom, quality-of life, and sleep outcomes as well as yoga or stretching/strengthening practice adherence and self-efficacy at home (questionnaires will be mailed in advance to participants at home, or can be completed via an on-line survey).
- Participants will be asked about their continued practice of yoga or stretching/strengthening techniques and their use of other strategies to manage their incontinence.
- Participants will be asked to mail back home practice diaries documenting their continued practice of yoga or stretching/strengthening techniques.
- Women will also be asked to complete a close-out satisfaction questionnaire addressing their overall satisfaction with study procedures and change in incontinence symptoms (questionnaires will be mailed in advance to participants at home, or can be completed via an on-line survey).

Recommended time windows for completion each of the post-randomization follow-up evaluations will be as follows:

Follow-Up Assessment	Time Window
2-week telephone call	8 to 20 days after the start of the yoga or physical conditioning program
6-week clinic visit	36 to 48 days (i.e., 6 weeks +/- 6 days) after

	the start of the yogaor physical conditioning program
9-week telephone call	57 to 69 days after the start of the yoga therapy or physical conditioning program
12-week clinic visit	78 to 90 days (i.e., 12 weeks +/- 6 days) after the start of the yogaor physical conditioning program
24-week (i.e., 12-week post-intervention) telephone and mail-in assessment	Within 189 days (27 weeks) of the start of the yoga or stretching/strengthening program (allowing time for return of mail-in packet)
36-week (i.e., 24-week post-intervention) telephone and mail-in assessment	Within 273 days (39 weeks) of the start of the yoga or stretching/strengthening program (allowing time for return of mail-in packet)

6.2.6 **Early Termination** (also see section below)

If a participant indicates that she needs or wishes to discontinue all participation in the study, then she will be encouraged to complete an Early Termination visit. This visit will include as many of the procedures originally scheduled to take place at the 12-Week Clinic Visit as possible, including: assessment of adverse events; re-administration of questionnaires about condition-specific quality of life, depression and anxiety symptoms, and yoga or stretching/strengthening self-efficacy; review of current medications; re-assessment of weight, blood pressure, and heart rate; re-assessment of physical function, autonomic function, and pelvic floor strength; and administration of a study satisfaction questionnaire. The participant's reasons for terminating participation early will be explored and documented in her file.

Of note, a participant can discontinue participation in study intervention classes or intervention home practice without discontinuing participation in the overall study. When possible, participants who decide to stop attending or engaging in the study intervention programs will be asked to provide data at originally planned outcome assessment timepoints (e.g., at 12 weeks and at 12 and 24 weeks after the originally planned end of the intervention program). In this case, no early termination visit will be performed, but planned outcome assessment visits will be completed.

.

6.2.7 Table of Measures and Procedures

Summary of Measures and Procedures at Study Visits									
	Screening Phone Call	Screening Clinic Visit	Baseline Clinic Visit	Randomization Phone Call	2/9-Week Phone Call	6-Week Clinic Visit	12-Week Clinic Visit	12 week post-intervention	24-week post-intervention
Brief telephone screening interview	X								
Informed consent		X							
Demographic/medical history questionnaires		X							
Health-related habits questionnaires		X							
Review of current medications		X				X	X	X	X
Basic physical mobility testing		X							
Height, weight, blood pressure, and pulse		X					X		
Urine dipstick testing		X							
Urine pregnancy testing (if appropriate)		X							
Fluid intake and behavior questionnaire			X			X	X	X	X
Voiding diary return and review			X			X	X	X	X
Review of eligibility and randomization			X*	X*					
Incontinence self-management pamphlet			X						
Assessment of credibility/expectations of treatment				X					
Incontinence Impact Questionnaire			X			X	X	X	X
Urogenital Distress Inventory-6			X			X	X	X	X
Patient Perception of Bladder Condition			X			X	X	X	X
PISQ-IR Sexual Function Questionnaire			X			X	X	X	X
Center for Epidemiologic Studies Depression Scale			X			X	X	X	X
Spielberg State-Trait Anxiety Inventory			X			X	X	X	X
Hospital Anxiety and Depression Scale			X			X	X	X	X
Perceived Stress Scale			X			X	X	X	X
Pittsburg Sleep Quality Index			X			X	X	X	X
Perceived physical function assessment			X			X	X	X	X
Physical performance testing			X			X	X		
Autonomic measures (RSA and PEP)			X				X		
Pelvic floor muscle strength assessment			X				X		
Adverse events assessment					X	X	X	X	X
Yoga/stretching posture/exercise self-efficacy				X		X	X		
Yoga/stretching practice self-efficacy questionnaires				X		X	X	X	X
Assessment of co-interventions						X	X	X	X
Satisfaction/close-out questionnaire							X		X

*Randomization will take place at either the Baseline Clinic Visit or the Randomization Phone Call

7. SAFETY ASSESSMENTS

7.1 Potential Risks and Protective Measures

Although participation in this study is not expected to place participants at substantial risk of harm, study procedures may be associated with the following risks:

- Yoga Intervention: Possible risks associated with group yoga instruction and home yoga practice include muscle soreness and muscle/ligament strain, or other musculoskeletal injury if participants fall or lose their balance while trying to practice yoga poses.
- Stretching/Strengthening Control Intervention: Similar to the yoga intervention, possible risks associated with the stretching/strengthening control intervention include muscle soreness and muscle/ligament strain, or musculoskeletal injury if participants fall or lose their balance while trying to learn or practice the stretching or strengthening exercises.
- Behavioral Incontinence Self-Management Pamphlet: Provision of standard written information on usual first-line behavioral self-management strategies for incontinence such as pelvic floor muscle exercises and timed urination should not pose any risks to participants, although participants may find it inconvenient or unpleasant to read the pamphlet.
- Questionnaires and Diaries: Although the information participants provide on data collection forms and diaries is confidential, some participants may feel embarrassed at having to answer questions, especially those related to urinary symptoms, depression, anxiety, or sexual function. There will be slight inconvenience in time and effort to complete diaries and questionnaires.
- Physical Exam Measurements: There are no direct risks associated with undergoing measurement of height, weight, blood pressure, or heart rate, although participants may experience this as inconvenient or unpleasant.
- Digital Pelvic Examination: Digital pelvic examination (not requiring a speculum) and assessment of pelvic floor muscle strength may be associated with discomfort or soreness in the perineal or vaginal area, emotional reaction to being touched in the genital area, feeling of fullness or pressure in the rectum, or urge to urinate during or following the exam.
- Perineometer Assessment: Similar to digital pelvic exam, perineometer assessments of pelvic floor muscle strength (at the Stanford site only0 may be associated with discomfort or soreness in the perineal or vaginal area, emotional reaction to being touched in the genital area, feeling of fullness or pressure in the rectum, or urge to urinate during or following the exam.
- Urinalysis and Urine Pregnancy Testing: There are no direct risks associated with collection of the clean-catch urine sample, although some participants may experience this as inconvenient or unpleasant.
- Heart Rate Variability and Impedance Cardiography: Heart rate variability and impedance cardiography will be obtained using a non-invasive Biopac electronic monitoring system. The only adverse effect that may result from these measurements is mild skin irritation at the site of the ECG or cardiac impedance

electrodes, which should disappear within 24 hours of removing the electrodes. Although noninvasive, measurement of heart rate variability and pre-ejection period may be experienced by participants as inconvenient or mildly unpleasant.

To minimize potential risks to participants associated with study interventions and procedures, the following steps will be taken:

- Yoga Intervention: The instructors leading the group yoga classes will be trained yoga teachers who will have experience in teaching adults from a wide range of ages to learn and practice yoga safely and will undergo specific, in-person training by the study's yoga expert consultants. The study protocol incorporates yoga props such as blocks and blankets to help participants perform yoga postures in such a way that will minimize the likelihood of muscle strain or musculoskeletal injury. The yoga instruction manuals and audio recordings are designed to include explicit tips on modifying or adapting postures to accommodate problems with mobility, flexibility, or balance.
- Stretching/Strengthening Intervention: Instruction for the control intervention classes will be provided by trained physical therapists or personal trainers who are experienced in teaching patients of all ages to perform physical exercises safely, and who will have completed in-person training with co-investigator Sarah Pawlowsky, PT. Stretching and strengthening exercises have been selected to be appropriate for middle-aged and older women who may have flexibility or mobility limitations in addition to incontinence.
- Behavioral Incontinence Self-Management Pamphlet: The pamphlet used in this trial has previously been administered and refined in other large-scale research studies. Study coordinators will be available to answer participants' questions about the pamphlet if they arise.
- Questionnaires and Diaries: Any paper-based questionnaires or diaries collected in this research will be stored in a locked file cabinet in a locked room in research facilities, and data obtained from diaries and questionnaires will be stored on password-secured servers. All study staff are fully trained in good clinical practice and Human Subject Projections procedures, and the importance of participant confidentiality will be emphasized.
- Physical Examination Measures: Study coordinators who are responsible for obtaining physical exam measures will have prior clinical experience with performing physical exam measurements in middle-aged and older women, and will receiving study-specific training to ensure that they perform measurements in a manner that is sensitive and minimizes discomfort to participants.
- Digital Pelvic Examination: Digital pelvic exams and assessment of pelvic floor muscle strength will be performed by clinically-trained consultants who have experience performing pelvic exams for clinical care and are trained to perform exams in a manner that is sensitive and minimizes discomfort to participants. Participants will be informed that they can ask to stop the exam for any reason; they can also decline the pelvic exam but take part in other aspects of the study.
- Perineometer Assessment: Perineometer-based assessment of pelvic floor muscle strength (at the Stanford site only) will be performed by a board-certified urogynecologist with experience performing pelvic exams for clinical care, experience in perineometry measurements, and training in performing exams in a manner that is sensitive and minimizes discomfort to participants. Participants

will be informed that they can ask to stop the assessment for any reason; they can also decline the pelvic exam but take part in other aspects of the study.

- Urinalysis and Pregnancy Testing: Study coordinators who are responsible for performing urine testing will be trained to instruct participants on providing urine samples in a manner that is sensitive and minimizes embarrassment.
- Heart Rate Variability and Impedance Cardiography: Participants with a history of allergy or adverse reaction to ECG electrodes or adhesives may to forgo undergo heart rate variability or impedance cardiography measures. If participants develop a skin reaction after testing is begun, measurements will be discontinued, and no further testing will be performed at any future visits.

7.2 Methods and Timing of Safety Assessments

To monitor participant safety, a clinical coordinator will assess for adverse events at each follow-up telephone and in-person contact following randomization, starting with the 2-Week Telephone Call. Additionally, participants will be given telephone numbers to call study staff in between scheduled study visits or calls to report any significant health changes.

Coordinators will not prompt participants to provide information about specific types of adverse events at follow-up visits, but instead encourage participants to volunteer information by asking the standardized, open-ended question, “Have there been any changes in your health since your last visit?” Any negative changes in health reported by participants will then be recorded on standardized AE or SAE forms, as appropriate (see below for definitions and documentation).

7.3 Adverse Events and Serious Adverse Events

7.3.1 AE/SAE Definitions

An Adverse Event (AE) is any untoward medical occurrence in participant that occurs during the study or with use of the study interventions. An adverse finding can include a sign, symptom, abnormal assessment, or any combination of these. Medical conditions or diseases present before starting study interventions will only be considered adverse events if they worsen after starting the intervention.

A Serious Adverse Event (SAE) is any AE that results in death, a life-threatening event, inpatient hospitalization or prolongation of existing hospitalization, a persistent or significant disability/incapacity, a congenital anomaly or birth defect, or any other important medical event that jeopardizes the safety of participants based upon appropriate medical judgment.

7.3.2 AE/SAE Documentation

All negative changes in health reported by participants will be recorded on standardized AE or SAE forms, as appropriate. Each adverse event will be assigned an AE number and will be recorded on a separate AE form to avoid duplication in reporting. For each AE, clinical coordinators will record the date of onset and resolution of the AE and record the nature or type of AE. In consultation with the principal investigator, coordinators will also indicate whether any indicate whether any specific action such as discontinuation of the study intervention was taken in response to the AE.

Using the Common Terminology Criteria for Adverse Events (CTCAE) system, all adverse events will be graded in severity using the 5-point CTCAE scale. According to this scale, AEs are considered grade 1 if they represent only mild symptoms or asymptomatic laboratory findings; grade 2 if they cause moderate symptoms that limit instrumental activities of daily living (shopping, transportation, household tasks); grade 3 if they are serious or disabling events or result in hospitalization or prolongation of hospitalization without being directly life-threatening; grade 4 if they are life-threatening events in which the participant is at risk of death at the time of the event if immediate intervention is not undertaken; or grade 5 if they are fatal adverse events.

Each serious adverse event will be assigned an SAE number and recorded on an SAE form. Similar to regular AEs, clinical coordinators will record the date of onset and resolution of each SAE and record the nature or type of SAE. Coordinators will also document any treatment provided for the SAE, indicate any laboratory or other clinical data obtained about the SAE, indicate the participant's condition at the time of reporting of the SAE, and indicate whether any specific action such as study discontinuation was taken in response to the SAE. Each SAE form will be reviewed and signed by the principal investigator, who will use a standard attribution scale to indicate the potential relationship between any SAEs and study interventions (not, unlikely, possible, probably, or definitely related to study intervention).

7.4 Reporting Procedures

7.4.1 SAE Reporting

- Clinical coordinators will notify the principal investigator immediately when a SAE is discovered, in addition to filling out a standardized SAE form that will be reviewed and signed by the principal investigator (see section above regarding SAE documentation).
- The principal investigator will then report any deaths to the institutional review board at the University of California San Francisco, the independent Data and Safety Monitor (DSM), and the NIDDK program officer within 24 hours of awareness of the event.
- Other unexpected serious adverse events that are potentially intervention-related will be reported by the principal investigator to the UCSF IRB, the DSM, and the NIDDK Program Officer within 48 hours of learning of the event.
- Anticipated or unrelated SAEs will be handled in a less urgent manner but will also be reported to the UCSF IRB and the DSM within 1 week of awareness of the event, and at least quarterly to the NIDDK Program Officer. Of note, no SAEs that are directly related to the study procedures are currently anticipated.

7.4.2 Non-Serious AE Reporting

- If a clinical coordinator discovers an AE that does not meet the definition of a SAE but may still pose a substantial risk of harm to a participant and is potentially associated with study interventions or procedures, he/she

will report the AE to the principal investigator within 1 working day, in addition to filling out a standardized AE form.

- Cumulative trends in all non-serious adverse events will be reviewed by the principal investigator and the DSM at scheduled DSM meetings (see below for DSM meeting schedule). More frequent cumulative review of non-serious adverse events may be performed if recommended by the DSM or desired by the principal investigator.
- Although the UCSF IRB does not require reporting of non-serious AEs to the UCSF IRB, the principal investigator may consult the UCSF IRB for input on handling of non-serious AEs if they may still pose a substantial risk of harm to a participant or are potentially associated with study interventions or procedures.

7.5 Follow-up for AEs/SAEs

From the time that they are first reported by participants until the end of the study, AEs and SAEs will be followed until resolved or considered stable. At each scheduled follow-up contact after initial discovery of an AE or SAE, a clinical coordinator will assess whether the AE or SAE is continuing or has resolved, and will update information about event status on the AE or SAE form, as appropriate. For SAEs, the clinical coordinator or principal investigator will also make more active attempts to communicate with participants in between scheduled study visits (with their permission) in order to monitor resolution of SAEs.

7.6 Independent Safety Monitoring

The conduct of the study and safety of participants will be evaluated by an independent Data and Safety Monitor (DSM), who will be a clinical researcher experienced with designing, implementing, and monitoring safety in therapeutic interventions studies as well as a practicing clinician. The DSM is independent of the investigators and staff participating in the study, and has no financial ties to the outcome of the study. The DSM will periodically review the conduct and outcomes of the study and provide feedback to the investigators, with particular attention to protecting the safety of the participants.

Prior to initiation of the trial, the DSM will review and approve the study design and plans for recruitment, adherence, interventions, data quality, and safety monitoring. Approximately twice a year after recruitment begins, the DSM will evaluate the adequacy and timeliness of participant recruitment, adherence to the protocol, and the potential of the study to meet the stated goals; evaluate the quality and integrity of the data, including adequacy of data management and data security procedures; evaluate participant safety including trends in adverse events and relationship to the study procedures; consider factors external to the study when relevant information, such as scientific developments, may have an impact on the safety of the participants or the ethical conduct of the study; and make recommendations, if necessary, to the investigators and the NIDDK on continuation, termination, or other modifications of the study protocol.

The DSM will periodically review aggregate trial data according to the Data and Safety Monitoring Plan (DSMP). An emergency meeting may also be called by the principal investigator at any time should questions of participant safety arise. Each review will include an assessment of the adequacy and timeliness of participant recruitment, adherence to the visit and intervention protocols, data quality and

timeliness, adverse effects, and participant safety. Interim reports for the DSM will be prepared by a biostatistician contracted by the Women's Health Clinical Research Center, and sent to the DSM at least 5 days prior to a pre-scheduled meeting or conference call.

After each interim review, the DSM will provide a signed statement that indicates whether the study should continue, terminate, or be altered based on ability to meet study recruitment and data quality goals and participant safety. He will include any recommendations for changes to the protocol if necessary to enhance participant safety or potentiate the ability of the trial to answer the research hypotheses. This statement will be provided to the principal investigator and will be sent to the UCSF IRB and to the NIDDK program officer. All materials, discussions, and proceedings of the DSM process will be completely confidential.

8. INTERVENTION DISCONTINUATION

Participation in the yoga and/or control interventions may be discontinued if determined to be necessary to protect the safety of a participant. The decision to discontinue participation in therapeutic interventions will be made on a case-by-case by the principal investigator, with input from the DSM if needed. Possible reasons for discontinuation of the therapeutic intervention may include: 1) development of a clinically significant adverse event limiting a participant's ability to safety take part in yoga or stretching/strengthening practice; 2) disruptive behavior exhibited by a participant during intervention classes or study visits that endangers the safety or comfort of other study participants, class instructors, or study staff; or 3) decision to terminate the study by the IRB, NIDDK, or other regulatory bodies. Participants may also decide that they want to discontinue participation in interventions, even if study staff urge them to complete assigned 12-week intervention programs.

In the event that participation in yoga or control intervention programs is discontinued, but the principal investigator and the participant feel it is safe, feasible, and appropriate for the participant to continue to provide outcomes data, the participant will continue to be followed through 36 weeks (until the original expected 24-week post-intervention assessment) to collect outcomes data. If follow-up assessments are continued, no modifications to the schedule of follow-up assessments will be made unless necessary to protect the safety of participants or to accommodate limitations in function resulting from an adverse event.

Follow-up assessments in participants who have discontinued the therapeutic interventions will not include continued evaluation of adherence to group classes or home practice, assessment of yoga posture or practice adherence self-efficacy, or independent evaluation of participants' success in performing yoga postures or stretching/strengthening exercises.

9. STATISTICAL CONSIDERATIONS

9.1 General Design Issues

A randomized, parallel-group, superiority trial design has been selected as the being the most appropriate design for the scientific goals of this research. Eligible participants will be randomly assigned in equal ratios to the yoga versus control interventions, with equivalent time and attention spent on study intervention and assessment procedures in both groups.

A non-specific muscle stretching-strengthening exercise program has been selected as an active behavioral control intervention for this trial. Although this control

intervention may offer non-specific benefits for participants' health, the investigators believe that it is unlikely to improve incontinence symptoms to the same extent as the yoga intervention. Inclusion of this plausible control intervention will also minimize differential expectations of treatment benefit between participants in the two groups, thus promoting similar adherence to interventions and visits in both groups.

9.2 Sample Size

Sample size has been calculated based on parameter estimates derived from both the investigators' past pilot studies on yoga as well as other group-based incontinence intervention trials conducted by study team members.[103] Assumptions include: a) average baseline frequency of incontinence of ~23-24 episodes/week; b) standard deviation (SD) of change in incontinence frequency of ~14.4; c) correlation between outcome values of ~0.59; and d) loss of ~15% by 3 months. Assuming a ~50% decrease in incontinence frequency among controls (which is the threshold at which women with daily incontinence start to perceive meaningful benefit from treatment[18]), the team will power the study to detect a between-group difference in reduction in incontinence of at least 20% in the yoga versus stretching/strengthening control group (corresponding to a ≥70% reduction in frequency of incontinence in the yoga group) at 3 months. Under these assumptions, a sample size of 240 (120 per group) will provide 80% power in 2-sided tests with 5% type-I error to detect a between-group difference in reduction in the primary outcome of total incontinence frequency of at least 20% in the yoga vs. control arm. With regard to secondary outcomes, this sample will also provide 80% power to detect moderate between-group differences of 0.26 to 0.39 SDs, assuming intraclass correlation of outcomes in the range of 0.4-0.8.

9.3 Randomization and Blinding

Randomization to the yoga or control intervention group will be performed by computer algorithm, with proof of eligibility required before assignment. Randomization will be implemented using randomly permuted blocks of sizes 2 and 4, and will also be stratified by clinical type of incontinence (i.e., stress-predominant, urgency-predominant), to ensure adequacy of randomization within types. To avoid manipulation, standard allocation concealment procedures will be followed.[102] An analyst will prepare sealed, opaque envelopes containing group assignment, numbered consecutively with the randomization sequence numbers. At the baseline visit, when eligibility and incontinence type have been confirmed, coordinators will enter the date, participant name, and study ID in the randomization log and select the next numbered envelope to determine group assignment. All envelopes will be retained for review; randomization dates and times should follow the order of the sequence numbers, providing a check on validity.

Due to the behavioral nature of the intervention, participants cannot be blinded to intervention assignment, nor can study staff responsible for administering interventions or monitoring adherence to interventions. However, study investigators and staff members who are not involved in administering interventions or monitoring adherence to interventions will be blinded.

Diary data used to evaluate primary and secondary efficacy outcomes will be abstracted by blinded analysts. Study-wide unblinding for the 12-week intervention period will take place when data from all dataforms and study measurements for the 12-week intervention period have been entered into the database for all participants,

data cleaning for this period has been completed, and the principal investigator has declared the 12-week study dataset to be complete. Unblinding of study investigators or staff prior to the study-wide unblinding date will be undertaken only in exceptional circumstances in which knowledge of intervention assignment is essential to guide treatment or to protect the safety of a participant.

9.4 Interim Analyses and Stopping Guidelines

Interim analyses and/or stopping for unexpected efficacy: Since the trial involves low-risk behavioral interventions that are of limited duration, the investigators believe that there is no scientific or ethical reason to stop the trial early or alter the trial design if treatment appears to be more effective than expected. Thus, as long as no safety issues arise, the trial will not be stopped or altered if the therapeutic intervention appears unexpectedly effective.

Interim analyses and/or stopping for harm: Although this study involves low-impact behavioral interventions that are not expected to pose a significant risk to participant safety, consideration may be given to stopping the trial early if unexpected and clinically significant adverse effects occur in either intervention group that may endanger the safety of participants. These may take the form of SAEs or non-serious AEs posing a substantial safety risk and warranting early termination of the trial. If evidence of adverse effects emerges, the investigators in consultation with the DSM may determine that these side effects are minor and the study can continue as planned, or may decide to alter or stop the study to prevent these side effects.

Interim analyses and stopping for futility: Consideration may be given to stopping the trial if recruitment and retention are so poor that the ability to meet recruitment goals or meet retention thresholds is severely compromised. For example, consideration will be given to stopping this study prior to its scheduled completion if recruitment falls more than 30% below planned rates and/or timely follow-up (i.e., completion of study visits within recommended windows) falls below 70%.

9.5 Outcomes

9.5.1 Primary Efficacy Outcome

The primary efficacy outcome will be change in the average frequency of any urinary incontinence over 12 weeks based on a validated 3-day voiding diary. To that end, frequency of incontinence will be assessed based on voiding diaries completed at screening/baseline, 6 weeks, and 12 weeks. If improvement in incontinence is observed over 12 weeks, additional analyses may explore persistence of benefits 12 and 24 weeks after the completion of the yoga or stretching/strengthening programs.

9.5.2 Secondary Efficacy Outcomes

Secondary efficacy outcomes will include change in the frequency of stress-type, urgency-type, daytime, and nighttime incontinence, also assessed using validated voiding diaries, over 12 weeks. The total number of any, stress-type, and urgency-type incontinence episodes will be calculated for each diary and then divided by the total number of days of recording to calculate the average number of incontinence episodes per day of each type.

Other secondary efficacy outcomes will include change in quality-of-life outcomes associated with incontinence over 12 weeks. To that end, changes

in depression and anxiety symptoms and incontinence-related quality of life will be assessed using validated questionnaires completed at screening/baseline, 6 weeks, and 12 weeks, as well as 12 and 24 weeks after the completion of yoga or control programs.

9.6 Data Analyses

9.6.1 Analyses to address main objectives

Objective 1: To determine the efficacy of a group-based yoga intervention in reducing the frequency of urinary incontinence in ambulatory midlife and older women.

Hypothesis 1A: Ambulatory incontinent women randomized to the yoga intervention will demonstrate at least a 20% greater decrease in total incontinence frequency (measured by validated voiding diaries) over 12 weeks, compared to women randomized to the control intervention.

Hypothesis 1B: If improvements in total incontinence frequency are observed at 12 weeks, these improvements will be sustained 12 and 24 weeks after the end of the intervention programs.

Planned analyses: Intervention effects on the primary outcome of total incontinence frequency will be estimated using a linear mixed model (LMM) for changes since baseline in the overall frequency of any incontinence episodes at 6 and 12 weeks, adjusting for baseline incontinence frequency. Analyses will be by intention to treat, according to intervention group assignment, and without regard to adherence to intervention classes or practice. The LMM will account for clustering using nested random effects for group intervention class series and individual participant. In addition to fixed effects for study location and type of incontinence, the LMM will include terms for intervention, time, and the time-by-intervention interaction, thus allowing for secular trends in controls, as well as different intervention effects at 6 and 12 weeks. The intervention effect will be summarized by an orthogonal contrast capturing any linear trend across 6 and 12 weeks; this makes use of information for participants with 6 but not 12 week outcomes. If the yoga intervention appears effective over 12 weeks, we will also assess maintenance of effects over time, by comparing changes since baseline at 12 weeks with those at 24 and 36 weeks, using an additional LMM. Intervention effects will again be summarized by an orthogonal contrast for linear trend across the three time points.

Objective 2: To determine the efficacy of a group-based yoga intervention in reducing the frequency of type- and context-specific urinary incontinence in ambulatory middle-aged and older women.

Hypothesis 2A: Ambulatory incontinent women randomized to the yoga intervention will report decreases in the frequency of stress-type, urgency-type, daytime, and nighttime incontinence (also measured by voiding diaries) over 12 weeks, compared to women randomized to the control intervention.

Hypothesis 2B: If improvements in stress-type, urgency-type, daytime, and nightime incontinence frequency are observed at 12 weeks, these improvements will be sustained 12 and 24 weeks after the end of the intervention programs.

Planned analyses: For additional confirmation of effects on the primary outcome of total incontinence frequency, we will examine effects on frequency of urgency

incontinence episodes and stress incontinence episodes, again using the modeling approach indicated above. Additional models will explore effects on frequency of daytime and nighttime incontinence.

Objective 3: To determine the efficacy of the yoga intervention in improving incontinence-related functioning and quality-of-life.

Hypothesis 3A: Ambulatory incontinent women randomized to the yoga intervention will report greater improvements in condition-specific functioning and quality of life (assessed by validated questionnaires) over 12 weeks, compared to women randomized to the control intervention.

Hypothesis 3B: Compared to women randomized to the control intervention, we hypothesize that women randomized to the yoga intervention will report sustained improvements in condition-specific quality of life 12 and 24 weeks after the end of the intervention programs.

Planned analyses: Additional LMMs will examine other secondary outcomes including incontinence-related quality of life as assessed by validated questionnaires (i.e., the Incontinence Impact Questionnaire (IIQ), Urogenital Distress Inventory (UDI), Patient Perception of Bladder Condition (PPBC), Pelvic Organ Prolapse/Incontinence Sexual Questionnaire, and IUGA-Revised (PISQ-IR) questionnaire), as well as depression and anxiety symptoms assessed by additional questionnaires (i.e., the Center for Epidemiology Studies Depression Scale (CES-D), Hospital and Anxiety Depression Scale (HADS), and State Trait Anxiety Inventory (STAI). We will again estimate intention-to-treat intervention effects, adjusting for study location, using the same modeling strategy as for objective 1. Transformation or categorization of questionnaire scores (in combination with logistic or proportional odds models) will be considered as needed to deal with skewness of outcomes.

Objective 4: To explore whether the yoga intervention is associated with improvements in 1) pelvic floor muscle strength, 2) peripheral autonomic function, and/or 3) lower extremity physical function, and assess whether improvements in these factors may mediate improvement in incontinence.

Hypothesis 4A: Among ambulatory incontinent women enrolled in this trial, the yoga intervention will be associated with improvements in a) pelvic floor muscle strength (assessed by pelvic exam), b) cardiac autonomic function (assessed by heart rate variability and impedance cardiography), and c) lower extremity physical function (assessed by physical performance assessment) over 12 weeks.

Hypothesis 4B: Among ambulatory incontinent women enrolled in this trial, changes in incontinence frequency over 12 weeks will be partly mediated by changes in a) pelvic floor muscle strength, b) cardiac autonomic function, and c) lower extremity physical function.

Planned analyses: Additional intermediary outcomes include pelvic floor muscle strength (measured by Brink scale), peripheral autonomic function (reflected by respiratory sinus arrhythmia [RSA] and cardiac pre-ejection period [PEP] parameters), and lower extremity physical function (assessed using the Short Physical Performance Battery [SBBP] and 2-minute step test). Intervention effects will first be estimated using LMMs, using the same modeling strategy used for objectives 1-3. Next, to assess for mediation of changes in incontinence frequency by changes in these factors, we will assess correlation between changes in these

parameters and change in incontinence frequency between baseline and 12 weeks. We will then examine attenuation of intervention effects estimates from the primary intention-to-treat analyses after adjustment for these potential mediators. Mediators will initially be analyzed as continuous variables (i.e., continuous change in RSA and PEP), but categorization will be considered in the event of skewed variable distributions. If evidence of mediation is observed, we will estimate 95% bias-corrected percentile confidence intervals confidence intervals for the proportion of intervention effect explained, using resampling by participant to fully account for the correlation between intervention effect estimates before and after adjustment for change in the mediators

9.6.2 Other analytic issues

Participant dropout (i.e., early termination or loss-to-follow-up). Participant dropout levels are expected to be <15% at 12 weeks of intervention, <20% at 12 weeks post-intervention follow-up, and <25% at 24 weeks post-intervention follow-up, assuming a similar rate of attrition over time. The assumption of non-informative dropout will be assessed by comparing baseline characteristics and early post-randomization outcomes of dropouts vs. non-dropouts. If dropout rates differ between groups, sensitivity analyses to evaluate the effects of participant dropout on estimates of intervention effects may be run using inverse probability of retention weights, which can legitimately include post-randomization variables associated with dropout. Alternately or in addition to this, we may use multiple imputation of missing outcomes under plausible informative missingness assumptions.[104]

Dose effects (length of exposure and intensity of practice). In the event that the primary analysis shows that yoga intervention is effective in reducing incontinence frequency over 12 weeks, we may use a closed test procedure to assess for dose response in length of intervention exposure without inflating type-I error.[105] Specifically, if we detect a statistically significant benefit over 12 weeks an alpha of 0.05, we may assess change from baseline to 6 weeks, and from 6 weeks to 12 weeks, also at alpha of 0.05, based on contrasts that can be obtained from the primary analysis model. In exploratory analyses, we may also examine whether cumulative hours spent in group intervention classes (based on class attendance logs), or total hours of home yoga or stretching/strengthening practice (documented by participants' home practice logs), are associated with changes in incontinence frequency or secondary outcomes in each group. After the end of the 12-week intervention, additional exploratory analyses may examine associations between continued yoga or control intervention practice (based on continued home practice hours) and maintenance of intervention effects.

Exploratory subgroup analyses. Intervention effects will be analyzed in subgroups in multivariate analysis based on a limited set of clinicopathologic factors that are hypothesized to have the potential to influence participants' response to the yoga and stretching/strengthening interventions. For example, we may explore whether baseline participant age, race/ethnicity, clinical type of incontinence, pelvic floor muscle strength, or physical function levels influence subsequent treatment-associated changes in primary and secondary outcomes. Subgroup-specific effects, estimated using the interaction model, will be presented only if the interaction between treatment and subgroup is statistically significant at $p<0.05$.

During the COVID-19 pandemic, intervention instruction in some study waves may be converted to remote platforms (i.e., interactive video-based class instruction rather than in-person class instruction). In that event, we will use the methods outlined above for exploratory subgroup analyses to evaluate differences in the effect of the intervention in waves that rely primarily on video-based instruction rather than in-person instruction.

Co-intervention effects. Because awareness of intervention assignment can affect use of co-interventions, women in both groups will be asked to refrain from using other clinical incontinence treatments during the study. Nevertheless, information about co-interventions will be collected at follow-up visits to permit assessment of potential effects of differential use of co-interventions on outcomes. To estimate the direct effect of the intervention in the absence of co-interventions, we may conduct additional analyses to add co-intervention use, as well as confounders of its effect on the outcome, to the primary treatment model; if factors that are also affected by the co-interventions are among the confounders, we may use inverse weighting rather than direct adjustment for this purpose.

10. DATA COLLECTION AND QUALITY ASSURANCE

10.1 Data Collection Forms

Study data will be obtained from participant-completed symptom diaries and logs, participant- or interviewer-administered questionnaires, physical examination and performance measures, and urine sampling/testing. Forms for recording or abstracting data will be developed specifically for this study, including:

- Voiding diary abstraction form: After participants complete their voiding diary at home, they will return the completed diary to the study clinic, and diary data will be abstracted using a Voiding Diary Abstraction Form. At the Baseline Visit, a clinical coordinator will abstract diary data for eligibility assessment purposes; at all post-randomization follow-up assessments, an analyst who is blinded to intervention assignment will abstract diary data.
- Questionnaires assessing depression and anxiety, condition-specific quality of life, and participant satisfaction: These questionnaire forms will be completed by the participant during clinic visits and returned in person to a clinical coordinator, or completed by the participant at home and then mailed back to the study clinic. Participants may enter questionnaire responses directly into the electronic study database using computer tablets or electronic questionnaire links. If questionnaires assessing study efficacy outcomes are completed by participants on paper after randomization and then entered by study staff, then the staff member responsible for data entry will be blinded to intervention assignment.
- Questionnaires assessing demographic history, general medical and urologic/urogynecologic history, medication use, tobacco and alcohol use, and physical activity and function: These questionnaires will be administered by a clinical coordinator during in-person study visits, and data from questionnaires will be entered by the coordinator into the electronic database. Because these data will not be used to assess efficacy outcomes, coordinators who administer and entering data from these forms will not necessarily be blinded to intervention assignment.

- Physical examination and urine testing data forms: Clinical coordinators will record the results of height and weight measurements, blood pressure and heart rate measurements, and urine dipstick and pregnancy testing on study-specific forms, and data from these forms will then be entered by coordinators into the electronic database. Because these data will not be used to assess any efficacy outcomes, the coordinators who are responsible for collecting, recording, and entering these data will not necessarily be blinded to intervention assignment.

10.2 Data Management

Data will be entered, managed, and edited using Medrio web-based Electronic Data Capture (EDC) software for Clinical Research, which is available for use without charge for investigator-initiated, university- or government-sponsored research. Medrio software meets the requirements for electronic records and signatures (21 CFR Part 11) and HIPAA. Data entered via Medrio can be accessed from machines on any network, can accommodate multiple users, allows for users at different sites to be issued differed password-protected logins, and includes tools to allow users to load data that collected offline.

10.3 Quality Assurance

10.3.1 Staff Training

All investigators, project directors, and clinical coordinators involved in the study will complete training in Human Subjects Research, which in most cases will be training offered by the Collaborative Institutional Training Initiative (CITI). CITI Human Subjects Research training includes specific modules on assessing risk to subjects, avoiding group harms, conflicts of interest, cultural competence, FDA-regulated research, HIPAA-regulated research, informed consent, IRB member responsibilities, IRB chair responsibilities, records-based research, research with vulnerable subjects, and unanticipated problems and reporting. All investigators, project directors, and coordinators will maintain active CITI certification during the study.

Coordinators and analysts involved in data collection will also attend a study-specific training meeting led by the principal investigator and the project directors prior to the start of participant recruitment. At a minimum, the training meeting will include: (1) an introduction to the goals, design, and procedures of the LILA study; (2) an overview of goals, structure, and procedures for administering and abstracting data from the voiding diary and other study-specific data collection measures; (3) a review of definitions and procedures for assessing, documenting, and reporting adverse events.

Coordinators and analysts will also receive training and undergo supervised practice in reviewing and abstracting data from the voiding diary as the primary efficacy measure in the trial. They will also receive training and undergo supervised practice performing physical exam measures, assessing physical function/performance, and obtaining autonomic function measurements. Following the training, clinical staff will be certified for performing these functions.

10.3.2 Data Quality Assessment

Data collection forms will be reviewed on an ongoing basis for data completeness and accuracy as well as protocol compliance. Forms used to collect data on the primary efficacy outcome (i.e., voiding diary abstraction forms) will be verified against paper-based source forms by a staff member who was not originally involved in entering the data.

Quality of data entry will be periodically assessed using measures such as number of missing diaries/forms and number of missing queries. The results of assessment of data quality will be incorporated into study progress reports for scheduled DSM meetings.

10.3.3 Protocol Deviations

Exceptions or deviations from the protocol are expected to occur rarely or not at all and, where possible, will be approved in advance by the principal investigator. Protocol exceptions/deviations may occur for the following reasons:

- exceptions necessary to protect the safety or well-being of a participant (in this case, the protocol exception should apply to that participant only)
- deviations due to oversight or error on the part of study staff, and subsequently detected by the investigators or study personnel.

For each protocol exception/deviation, study staff will document the event on a Protocol Deviations Log (located in the Regulatory Binder). Entries in the Protocol Deviations Log should include the participant ID, date of the deviation, the date the deviation is recorded, description of the deviation, and action taken in response to the deviation, if any. The principal investigator will document approval for each deviation determined in advance, or acknowledgement for each deviation detected after the fact.

For this study, missed yoga or control intervention classes will not be considered a protocol deviation, and neither will failure to complete recommended home practice sessions, although study staff will make every effort to promote adherence to classes and home practice (see adherence section above).

10.3.4 External Monitoring

If desired by NIDDK, the study team will undergo on-site monitoring by an independent quality monitor who is independent of both the sponsor and UCSF. Site monitoring visits may include review of participant records, informed consent forms, source dataforms, and the electronic study database. The schedule of site monitoring will be agreed upon in advance by the NIDDK, the principal investigator, and the independent monitoring organization. The principal investigator, project manager, and study coordinators will be available to meet in person with the external monitor and provide access to study-specific forms, materials, and databases.

11. PARTICIPANT RIGHTS AND CONFIDENTIALITY

11.1 Institutional Review Board (IRB) Review

The study protocol, informed consent document, data and safety monitoring plan, and data collection forms will be reviewed and approved by the UCSF IRB prior to implementation of study procedures. Any subsequent modifications to these documents will also be reviewed and approved by the UCSF IRB prior to administration in the study.

11.2 Informed Consent Forms

A signed consent form will be obtained from each participant at the Screening Visit before in-person data collection procedures are initiated (see section on informed consent procedures). A copy of the consent form will be given to each participant, and documentation of signed consent will be filed in the participant's study file.

11.3 Participant Confidentiality

Study data will be protected to preserve participant confidentiality both during and after the study. Each participant will be assigned a unique numerical study identifier which will be used on study forms instead of names or other identifying information. Only the study coordinators or investigators who need to get access to identifiers in order to contact participants will have access to the document linking study IDs to participant identifiers. Any paper source forms will be stored in locked cabinets in locked study offices, and only research personnel who need to access these forms for data collection, editing, or safety/quality monitoring will have access to them. Information that could identify individual participants will not be released without written permission of the participant, except as necessary for monitoring by the IRB, NIDDK, OHRP, or other agencies responsible for protecting participant safety.

11.4 Study Discontinuation

The study may be discontinued by the Steering Committee at any time at the recommendation of the UCSF IRB, the NIDDK, the OHRP, or other government agencies if necessary to protect the safety or confidentiality of research participants.

12. ETHICAL CONSIDERATIONS

This research will be conducted in accordance with principles outlined in, "The Belmont Report: Ethical Principles and Guidelines for the Protection of Human Subjects in Research" (National Commission for the Protection of Human Subjects of Biomedical and Behavioral Research, April 1979), which include respect for persons, beneficence, and justice.

13. STEERING/PUBLICATIONS COMMITTEE

Conduct of this research will be overseen by a Steering Committee composed of the principal investigator Alison Huang, MD, MAS and co-investigators Leslee Subak, MD and Margaret Chesney, PhD. Publication of the results of this trial will be governed by the policies and procedures developed by the Steering Committee.

14. PUBLICATION OF RESEARCH FINDINGS

Any publications resulting from this research will be made available to the public, in accordance with NIH public access policy. At the request of the NIDDK program officer, the principal investigator will also provide a copy of any abstracts or manuscripts resulting from this work to the program officer prior to submission.

15. REFERENCES

1. Melville JL, Katon W, Delaney K, Newton K. Urinary incontinence in US women: a population-based study. *Arch Intern Med.* 2005;165(5):537-542.
2. Thom DH, Haan MN, Van Den Eeden SK. Medically recognized urinary incontinence and risks of hospitalization, nursing home admission and mortality. *Age Ageing.* 1997;26(5):367-374.
3. Langa KM, Fultz NH, Saint S, Kabeto MU, Herzog AR. Informal caregiving time and costs for urinary incontinence in older individuals in the United States. *J Am Geriatr Soc.* 2002;50(4):733-737.
4. Fultz NH, Fisher GG, Jenkins KR. Does urinary incontinence affect middle-aged and older women's time use and activity patterns? *Obstet Gynecol.* 2004;104(6):1327-1334.
5. Huang AJ, Brown JS, Thom DH, Fink HA, Yaffe K. Urinary incontinence in older community-dwelling women: the role of cognitive and physical function decline. *Obstet Gynecol.* 2007;109(4):909-916.
6. Sampselle CM, Harlow SD, Skurnick J, Brubaker L, Bondarenko I. Urinary incontinence predictors and life impact in ethnically diverse perimenopausal women. *Obstet Gynecol.* 2002;100(6):1230-1238.
7. Bogner HR. Urinary incontinence and psychological distress in community-dwelling older African Americans and whites. *J Am Geriatr Soc.* 2004;52(11):1870-1874.
8. Brown JS, Vittinghoff E, Wyman JF, et al. Urinary incontinence: does it increase risk for falls and fractures? Study of Osteoporotic Fractures Research Group. *J Am Geriatr Soc.* 2000;48(7):721-725.
9. Wilson L, Brown JS, Shin GP, Luc KO, Subak LL. Annual direct cost of urinary incontinence. *Obstet Gynecol.* 2001;98(3):398-406.
10. Bump RC, Hurt WG, Fantl JA, Wyman JF. Assessment of Kegel pelvic muscle exercise performance after brief verbal instruction. *Am J Obstet Gynecol.* 1991;165(2):322-327; discussion 327-329.
11. Borello-France D, Burgio KL, Goode PS, et al. Adherence to behavioral interventions for urge incontinence when combined with drug therapy: adherence rates, barriers, and predictors. *Physical therapy.* 2010;90(10):1493-1505.
12. Milne JL, Moore KN. Factors impacting self-care for urinary incontinence. *Urol Nurs.* 2006;26(1):41-51.
13. Borello-France D, Burgio KL, Goode PS, et al. Adherence to behavioral interventions for stress incontinence: rates, barriers, and predictors. *Physical therapy.* 2013;93(6):757-773.
14. Imamura M, Abrams P, Bain C, et al. Systematic review and economic modelling of the effectiveness and cost-effectiveness of non-surgical treatments for women with stress urinary incontinence. *Health technology assessment (Winchester, England).* 2010;14(40):1-188, iii-iv.
15. Washington BB, Raker CA, Sung VW. Barriers to pelvic floor physical therapy utilization for treatment of female urinary incontinence. *Am J Obstet Gynecol.* 2011;205(2):152 e151-159.

16. Gopal M, Haynes K, Bellamy SL, Arya LA. Discontinuation rates of anticholinergic medications used for the treatment of lower urinary tract symptoms. *Obstet Gynecol.* 2008;112(6):1311-1318.
17. Diokno A, Yuhico M, Jr. Preference, compliance and initial outcome of therapeutic options chosen by female patients with urinary incontinence. *J Urol.* 1995;154(5):1727-1730; discussion 1731.
18. Shamliyan T, Wyman J, Kane RL. *Nonsurgical Treatments for Urinary Incontinence in Adult Women: Diagnosis and Comparative Effectiveness.* Rockville MD2012.
19. Sultana CJ, Campbell JW, Pisanelli WS, Sivinski L, Rimm AA. Morbidity and mortality of incontinence surgery in elderly women: an analysis of Medicare data. *Am J Obstet Gynecol.* 1997;176(2):344-348.
20. Im HW, Kim MD, Kim JC, Choi JB. Autonomous nervous system activity in women with detrusor overactivity. *Korean J Urol.* 2010;51(3):183-186.
21. Choi JB, Kim YB, Kim BT, Kim YS. Analysis of heart rate variability in female patients with overactive bladder. *Urology.* 2005;65(6):1109-1112; discussion 1113.
22. Hubeaux K, Deffieux X, Ismael SS, Raibaut P, Amarenco G. Autonomic nervous system activity during bladder filling assessed by heart rate variability analysis in women with idiopathic overactive bladder syndrome or stress urinary incontinence. *J Urol.* 2007;178(6):2483-2487.
23. Fazeli MS, Collet JP, MacNeily AE, Afshar K. Cardiac Autonomic Nervous System Activity in Children with Bladder and Bowel Dysfunction. *J Urol.* 2016;195(4 Pt 2):1245-1249.
24. Hotaling JM, Sarma AV, Patel DP, et al. Cardiovascular Autonomic Neuropathy, Sexual Dysfunction, and Urinary Incontinence in Women With Type 1 Diabetes. *Diabetes care.* 2016;39(9):1587-1593.
25. Hsiao SM, Su TC, Chen CH, Chang TC, Lin HH. Autonomic dysfunction and arterial stiffness in female overactive bladder patients and antimuscarinics related effects. *Maturitas.* 2014;79(1):65-69.
26. Telles S, Nagarathna R, Nagendra HR, Desiraju T. Physiological changes in sports teachers following 3 months of training in Yoga. *Indian J Med Sci.* 1993;47(10):235-238.
27. Udupa K, Madanmohan, Bhavanani AB, Vijayalakshmi P, Krishnamurthy N. Effect of pranayam training on cardiac function in normal young volunteers. *Indian J Physiol Pharmacol.* 2003;47(1):27-33.
28. Vempati RP, Telles S. Yoga-based guided relaxation reduces sympathetic activity judged from baseline levels. *Psychol Rep.* 2002;90(2):487-494.
29. Damodaran A, Malathi A, Patil N, Shah N, Suryavansihi, Marathe S. Therapeutic potential of yoga practices in modifying cardiovascular risk profile in middle aged men and women. *J Assoc Physicians India.* 2002;50(5):633-640.
30. Brown JS, Seeley DG, Fong J, Black DM, Ensrud KE, Grady D. Urinary incontinence in older women: who is at risk? Study of Osteoporotic Fractures Research Group. *Obstet Gynecol.* 1996;87(5 Pt 1):715-721.
31. Tinetti ME, Inouye SK, Gill TM, Doucette JT. Shared risk factors for falls, incontinence, and functional dependence. Unifying the approach to geriatric syndromes. *Jama.* 1995;273(17):1348-1353.
32. Fritel X, Lachal L, Cassou B, Fauconnier A, Dargent-Molina P. Mobility impairment is associated with urge but not stress urinary incontinence in community-dwelling older women: results from the Ossebo study. *BJOG : an international journal of obstetrics and gynaecology.* 2013;120(12):1566-1572.
33. Suskind AM, Cawthon PM, Nakagawa S, et al. Urinary Incontinence in Older Women: The Role of Body Composition and Muscle Strength: From the Health, Aging, and Body Composition Study. *Journal of the American Geriatrics Society.* 2017;65(1):42-50.

34. Kim H, Yoshida H, Suzuki T. The effects of multidimensional exercise on functional decline, urinary incontinence, and fear of falling in community-dwelling elderly women with multiple symptoms of geriatric syndrome: a randomized controlled and 6-month follow-up trial. *Archives of gerontology and geriatrics.* 2011;52(1):99-105.
35. Kim H, Yoshida H, Suzuki T. Effects of exercise treatment with or without heat and steam generating sheet on urine loss in community-dwelling Japanese elderly women with urinary incontinence. *Geriatrics & gerontology international.* 2011;11(4):452-459.
36. Banasik J, Williams H, Haberman M, Blank SE, Bendel R. Effect of Iyengar yoga practice on fatigue and diurnal salivary cortisol concentration in breast cancer survivors. *J Am Acad Nurse Pract.* 2011;23(3):135-142.
37. Beddoe AE, Paul Yang CP, Kennedy HP, Weiss SJ, Lee KA. The effects of mindfulness-based yoga during pregnancy on maternal psychological and physical distress. *Journal of obstetric, gynecologic, and neonatal nursing : JOGNN / NAACOG.* 2009;38(3):310-319.
38. Evans S, Cousins L, Tsao JC, Sternlieb B, Zeltzer LK. Protocol for a randomized controlled study of Iyengar yoga for youth with irritable bowel syndrome. *Trials.* 2011;12:15.
39. Evans S, Cousins L, Tsao JC, Subramanian S, Sternlieb B, Zeltzer LK. A randomized controlled trial examining Iyengar yoga for young adults with rheumatoid arthritis: a study protocol. *Trials.* 2011;12:19.
40. Williams K, Abildso C, Steinberg L, et al. Evaluation of the effectiveness and efficacy of Iyengar yoga therapy on chronic low back pain. *Spine.* 2009;34(19):2066-2076.
41. Williams KA, Petronis J, Smith D, et al. Effect of Iyengar yoga therapy for chronic low back pain. *Pain.* 2005;115(1-2):107-117.
42. Kolasinski SL, Garfinkel M, Tsai AG, Matz W, Van Dyke A, Schumacher HR. Iyengar yoga for treating symptoms of osteoarthritis of the knees: a pilot study. *J Altern Complement Med.* 2005;11(4):689-693.
43. Michalsen A, Grossman P, Acil A, et al. Rapid stress reduction and anxiolysis among distressed women as a consequence of a three-month intensive yoga program. *Med Sci Monit.* 2005;11(12):CR555-561.
44. Oken BS, Kishiyama S, Zajdel D, et al. Randomized controlled trial of yoga and exercise in multiple sclerosis. *Neurology.* 2004;62(11):2058-2064.
45. Huang AJ, Grady D, Appa A, Subak LL. A pilot randomized trial of a behavioral slow-breathing intervention to treat urgency incontinence in women. American Geriatrics Society Annual Scientific Meeting; May 4, 2012, 2012; Seattle, Washington.
46. Locher JL, Goode PS, Roth DL, Worrell RL, Burgio KL. Reliability assessment of the bladder diary for urinary incontinence in older women. *J Gerontol A Biol Sci Med Sci.* 2001;56(1):M32-35.
47. Brown JS, McNaughton KS, Wyman JF, et al. Measurement characteristics of a voiding diary for use by men and women with overactive bladder. *Urology.* 2003;61(4):802-809.
48. Shumaker SA, Wyman JF, Uebersax JS, McClish D, Fantl JA. Health-related quality of life measures for women with urinary incontinence: the Incontinence Impact Questionnaire and the Urogenital Distress Inventory. Continence Program in Women (CPW) Research Group. *Qual Life Res.* 1994;3(5):291-306.
49. Uebersax JS, Wyman JF, Shumaker SA, McClish DK, Fantl JA. Short forms to assess life quality and symptom distress for urinary incontinence in women: the Incontinence Impact Questionnaire and the Urogenital Distress Inventory. Continence Program for Women Research Group. *Neurourol Urodyn.* 1995;14(2):131-139.
50. Coyne KS, Matza LS, Kopp Z, Abrams P. The validation of the patient perception of bladder condition (PPBC): a single-item global measure for patients with overactive bladder. *Eur Urol.* 2006;49(6):1079-1086.

51. Matza LS, Thompson CL, Krasnow J, Brewster-Jordan J, Zyczynski T, Coyne KS. Test-retest reliability of four questionnaires for patients with overactive bladder: the overactive bladder questionnaire (OAB-q), patient perception of bladder condition (PPBC), urgency questionnaire (UQ), and the primary OAB symptom questionnaire (POSQ). *Neurourol Urodyn.* 2005;24(3):215-225.
52. Rogers RG, Rockwood TH, Constantine ML, et al. A new measure of sexual function in women with pelvic floor disorders (PFD): the Pelvic Organ Prolapse/Incontinence Sexual Questionnaire, IUGA-Revised (PISQ-IR). *International urogynecology journal.* 2013;24(7):1091-1103.
53. WW E, C M, C S, A T, M Y. Center for Epidemiologic Studies Depression Scale: Review and revision (CESD and CESD-R). In: ME M, ed. *The Use of Psychological Testing for Treatment Planning and Outcomes Assessment.* 3rd ed. Mahwah, NJ: Lawrence Erlbaum; 2004:363-377.
54. Barnes L, Harp D, Jung W. Reliability Generalization of Score son the Spielberger State-Trait Anxiety Inventory. *Educational and Psychological Measurement.* 2002;62(4):603-618.
55. Quek KF, Low WY, Razack AH, Loh CS, Chua CB. Reliability and validity of the Spielberger State-Trait Anxiety Inventory (STAI) among urological patients: a Malaysian study. *Med J Malaysia.* 2004;59(2):258-267.
56. Zigmond AS, Snaith RP. The hospital anxiety and depression scale. *Acta Psychiatr Scand.* 1983;67(6):361-370.
57. Rogers R, Bachmann G, Jumadilova Z, et al. Efficacy of tolterodine on overactive bladder symptoms and sexual and emotional quality of life in sexually active women. *Int Urogynecol J Pelvic Floor Dysfunct.* 2008;19(11):1551-1557.
58. Cohen S, Williamson G. Perceived stress in a probability sample of the United States. In: Sapacapam S, Oskamp S, eds. *The social psychology of health: Claremont Symposium on applied psychology.* Newbury Park, CA: Sage; 1988.
59. Junkin S. *Yoga and self-esteem: exploring change in middle-aged women*. Saskatoon: Kinesiology, University of Saskatchewan; 2007.
60. Marcus BH, Selby VC, Niaura RS, Rossi JS. Self-efficacy and the stages of exercise behavior change. *Res Q Exerc Sport.* 1992;63(1):60-66.
61. Borkovec T, SD N. Credibility of analogue therapy rationales. *Journal of Behavior Therapy and Experimental Psychiatry.* 1972;3:257-260.
62. Rose M, Bjorner JB, Becker J, Fries JF, Ware JE. Evaluation of a preliminary physical function item bank supported the expected advantages of the Patient-Reported Outcomes Measurement Information System (PROMIS). *J Clin Epidemiol.* 2008;61(1):17-33.
63. Springer BA, Marin R, Cyhan T, Roberts H, Gill NW. Normative values for the unipedal stance test with eyes open and closed. *Journal of geriatric physical therapy (2001).* 2007;30(1):8-15.
64. Stel VS, Smit JH, Pluijm SM, Lips P. Balance and mobility performance as treatable risk factors for recurrent falling in older persons. *Journal of clinical epidemiology.* 2003;56(7):659-668.
65. Jones CJ, Rikli RE, Beam WC. A 30-s chair-stand test as a measure of lower body strength in community-residing older adults. *Research quarterly for exercise and sport.* 1999;70(2):113-119.
66. Guralnik JM, Simonsick EM, Ferrucci L, et al. A short physical performance battery assessing lower extremity function: association with self-reported disability and prediction of mortality and nursing home admission. *J Gerontol.* 1994;49(2):M85-94.

67. Pahor M, Guralnik JM, Ambrosius WT, et al. Effect of structured physical activity on prevention of major mobility disability in older adults: the LIFE study randomized clinical trial. *Jama.* 2014;311(23):2387-2396.
68. Perera S, Studenski S, Newman A, et al. Are estimates of meaningful decline in mobility performance consistent among clinically important subgroups? (Health ABC study). *J Gerontol A Biol Sci Med Sci.* 2014;69(10):1260-1268.
69. Jones CJ, Rikli RE. Measuring functional fitness of older adults. *The Journal on Active Aging.* 2002:24-30.
70. Rikli RE, Jones CJ. Development and validation of criterion-referenced clinically relevant fitness standards for maintaining physical independence in later years. *Gerontologist.* 2013;53(2):255-267.
71. Berntson GG, Bigger JT, Jr., Eckberg DL, et al. Heart rate variability: origins, methods, and interpretive caveats. *Psychophysiology.* 1997;34(6):623-648.
72. Berntson GG, Cacioppo JT, Binkley PF, Uchino BN, Quigley KS, Fieldstone A. Autonomic cardiac control. III. Psychological stress and cardiac response in autonomic space as revealed by pharmacological blockades. *Psychophysiology.* 1994;31(6):599-608.
73. Mendes WB. Assessing Autonomic Nervous System Activity. In: Harmon-Jones E, Beer J, eds. *Methods in Social Neuroscience.* New York: The Guilford Press; 2009:118-147.
74. Blascovish J, Vanman E, Mendes WB, Dickerson S. Autonomic Nervous System: Obtaining, Quantifying, and Interpreting Peripheral Physiological Responses In: *Social Psychophysiology for Social and Personality Psychology.* London: SAGE Publications, Inc.; 2011:10-40.
75. Mendes WB. Assessing the autonomic nervous system. In: *Methods in social neuroscience.* New York: Guilford Press; 2009:118-147.
76. Mendes WB. Inspired by the question not the measure: Exploiting neurobiological responses in the service of intergroup research. In: Ellemers N, Scheepers D, Derks B, eds. *Social Neuroscience of Prejudice.* New York: Psychology Press; 2013.
77. Mendes WB, Park J. Neurobiological concomitants of motivation states. In: Elliot AJ, ed. *Advances in Motivational Science.* Waltham, MA: Academic Press; 2014:233-270.
78. Mendes WB. Emotion and Autonomic Nervous System Concomitants. In: Barrett LF, Haviland-Jones J, Lewis J, eds. *Handbook of Emotions, 4th Edition.* New York: Guildford Publications, Inc.; 2015 (in press).
79. Gibson CJ, Mendes WB, Schembri M, Grady D, Huang AJ. Cardiac autonomic function and hot flashes among perimenopausal and postmenopausal women. *Menopause (New York, NY).* 2017;24(7):756-761.
80. Task Force of the European Society of Cardiology and the North American Society of Pacing and Electrophysiology. Heart rate variability; standards of measurement, physiological interpretation and clinical use. *Circulation.* 1996;93:1043-1065.
81. Cavanagh P, Alvarez GA. Tracking multiple targets with multifocal attention. *Trends in cognitive sciences.* 2005;9(7):349-354.
82. Sherwood A, Allen MT, Fahrenberg J, Kelsey RM, Lovallo WR, van Doornen LJ. Methodological guidelines for impedance cardiography. *Psychophysiology.* 1990;27(1):1-23.
83. Akinola M, Mendes WB. It's good to be the king: Neurobiological benefits of higher social standing. *Social and Personality Psychological Science.* 2014;5:43-51.
84. Borello-France DF, Downey PA, Zyczynski HM, Rause CR. Continence and quality-of-life outcomes 6 months following an intensive pelvic-floor muscle exercise program for female stress urinary incontinence: a randomized trial comparing low- and high-frequency maintenance exercise. *Phys Ther.* 2008;88(12):1545-1553.

85. Borello-France DF, Handa VL, Brown MB, et al. Pelvic-floor muscle function in women with pelvic organ prolapse. *Phys Ther.* 2007;87(4):399-407.
86. Brink CA, Wells TJ, Sampselle CM, Taillie ER, Mayer R. A digital test for pelvic muscle strength in women with urinary incontinence. *Nurs Res.* 1994;43(6):352-356.
87. FitzGerald MP, Burgio KL, Borello-France DF, et al. Pelvic-floor strength in women with incontinence as assessed by the brink scale. *Phys Ther.* 2007;87(10):1316-1324.
88. Hundley AF, Wu JM, Visco AG. A comparison of perineometer to brink score for assessment of pelvic floor muscle strength. *American journal of obstetrics and gynecology.* 2005;192(5):1583-1591.
89. King VG, Boyles SH, Worstell TR, Zia J, Clark AL, Gregory WT. Using the Brink score to predict postpartum anal incontinence. *American journal of obstetrics and gynecology.* 2010;203(5):486 e481-485.
90. Mayer R, Wells TJ, Brink CA, Clark P. Correlations between dynamic urethral profilometry and perivaginal pelvic muscle activity. *Neurourology and urodynamics.* 1994;13(3):227-235.
91. Andresen EM, Fitch CA, McLendon PM, Meyers AR. Reliability and validity of disability questions for US Census 2000. *Am J Public Health.* 2000;90(8):1297-1299.
92. Arya LA, Banks C, Gopal M, Northington GM. Development and testing of a new instrument to measure fluid intake, output, and urinary symptoms: the questionnaire-based voiding diary. *Am J Obstet Gynecol.* 2008;198(5):559.e551-557.
93. Arya LA, Heidi H, Cory L, Segal S, Northington GM. Construct validity of a questionnaire to measure the type of fluid intake and type of urinary incontinence. *Neurourol Urodyn.* 2011;30(8):1597-1602.
94. Segal S, Saks EK, Arya LA. Self-assessment of fluid intake behavior in women with urinary incontinence. *J Womens Health (Larchmt).* 2011;20(12):1917-1921.
95. Booth M. Assessment of physical activity: an international perspective. *Res Q Exerc Sport.* 2000;71(2 Suppl):S114-120.
96. Craig CL, Marshall AL, Sjostrom M, et al. International physical activity questionnaire: 12-country reliability and validity. *Med Sci Sports Exerc.* 2003;35(8):1381-1395.
97. Buysse DJ, Reynolds CF, 3rd, Monk TH, Berman SR, Kupfer DJ. The Pittsburgh Sleep Quality Index: a new instrument for psychiatric practice and research. *Psychiatry Res.* 1989;28(2):193-213.
98. Buysse DJ, Reynolds CF, 3rd, Monk TH, Hoch CC, Yeager AL, Kupfer DJ. Quantification of subjective sleep quality in healthy elderly men and women using the Pittsburgh Sleep Quality Index (PSQI). *Sleep.* 1991;14(4):331-338.
99. Hoch CC, Dew MA, Reynolds CF, 3rd, et al. A longitudinal study of laboratory- and diary-based sleep measures in healthy "old old" and "young old" volunteers. *Sleep.* 1994;17(6):489-496.
100. Monk TH, Reynolds CF, Kupfer DJ, et al. The Pittsburgh Sleep Diary. *Journal of sleep research.* 1994;3(2):111-120.
101. Buysse DJ, Germain A, Moul DE, et al. Efficacy of brief behavioral treatment for chronic insomnia in older adults. *Archives of internal medicine.* 2011;171(10):887-895.
102. Schulz KF, Grimes DA. Allocation concealment in randomised trials: defending against deciphering. *Lancet.* 2002;359(9306):614-618.
103. Subak LL, Wing R, West DS, et al. Weight loss to treat urinary incontinence in overweight and obese women. *N Engl J Med.* 2009;360(5):481-490.
104. Schafer JL. Multiple imputation: a primer. *Stat Methods Med Res.* 1999;8(1):3-15.
105. Rom DM, Costello RJ, Connell LT. On closed test procedures for dose-response analysis. *Stat Med.* 1994;13(15):1583-1596.
