# Supplementary material for: Effects of Hatha Yoga vs Physical Conditioning on Sleep in Women With Urinary Incontinence: A Secondary Analysis of a Randomized Clinical Trial
Source: JAMA Netw Open. 2025 Dec 8;8(12):e2546499. doi: 10.1001/jamanetworkopen.2025.46499 (PMC12687092; doi:10.1001/jamanetworkopen.2025.46499)
Supplement: Supplement 2. — eTable. Prospective associations between change in nocturnal urinary incontinence and nocturnal voiding and change in sleep outcomes from baseline to 6 and 12 weeks in the combined-group sample [file jamanetwopen-e2546499-s002.pdf]

Supplemental Online Content

Hough E, Goldstein LA, Subak LL, et al. Effects of hatha yoga vs physical conditioning on sleep in women with urinary incontinence: a secondary analysis of a randomized clinical trial. *JAMA Netw Open*. 2025;8(12):e2546499. doi:10.1001/jamanetworkopen.2025.46499

eTable. Prospective associations between change in nocturnal urinary incontinence and nocturnal voiding and change in sleep outcomes from baseline to 6 and 12 weeks in the combined-group sample

This supplemental material has been provided by the authors to give readers additional information about their work.

© 2025 Hough E et al. *JAMA Network Open*.

eTable. Prospective associations between change in nocturnal urinary incontinence and nocturnal voiding and change in sleep outcomes from baseline to 6 and 12 weeks in the combined-group sample

**Change relative to each 1-episode decrease in nightly urinary incontinence frequency**			
	**Number of Participants (N)**	**Beta Coefficient (Std err)**	**P-value**
Pittsburgh Sleep Quality Index (PSQI) global score change	216	0.43 (0.24)	0.08
Total sleep time change (minutes)	216	-4.19 (6.77)	0.54
[a]Wake after sleep onset (WASO) change (minutes)	215	-0.76 (2.45)	0.76
[a]Sleep efficiency change (percent increase)	216	-0.04 (0.05)	0.34
**Change relative to each 1-episode decrease in nightly voiding in the toilet**			
Pittsburgh Sleep Quality Index (PSQI) global score change	216	0.09 (0.15)	0.54
Total sleep time change (minutes)	216	3.54 (3.99)	0.38
[a]Wake after sleep onset (WASO) change (minutes)	215	2.71 (1.47)	0.07
[a]Sleep efficiency change (percent increase)	216	-0.04 (0.03)	0.18

Beta coefficients and p-values were derived from linear regression analyses, adjusted for study visit and intervention group, as well as for predominant incontinence type and study site (as original randomization stratification factors).
[a]Sleep efficiency and WASO data were winsorized to address skewed data distributions.

© 2025 Hough E et al. *JAMA Network Open*.
