# Supplementary material for: Effects of Hatha Yoga vs Physical Conditioning on Sleep in Women With Urinary Incontinence: A Secondary Analysis of a Randomized Clinical Trial
Source: JAMA Netw Open. 2025 Dec 8;8(12):e2546499. doi: 10.1001/jamanetworkopen.2025.46499 (PMC12687092; doi:10.1001/jamanetworkopen.2025.46499)
Supplement: Supplement 3. — Data Sharing Statement [file jamanetwopen-e2546499-s003.pdf]

Data Sharing Statement

Hough. Effects of Hatha Yoga vs Physical Conditioning on Sleep in Women With Urinary Incontinence: A Randomized Clinical Trial. *JAMA Netw Open*. Published online December 8, 2025. doi:10.1001/jamanetworkopen.2025.46499

Data
Additional Information: Ancillary Study of the Lessening Incontinence With Low-impact Activity Study, https://clinicaltrials.gov/study/NCT04776720 , NCT04776720
Data available: Yes
Data types: Deidentified participant data, Data dictionary
How to access data: Please contact Michael.Schembri@ucsf.edu for Data Sharing requests. **When available:** With publication

Supporting Documents
Document types: None

Additional Information
Who can access the data: Data will be made available to individuals who complete a data request and sign a data-sharing agreement, subject to UCSF policies.
Types of analyses: Any purpose
Mechanisms of data availability: Individuals requesting access to data will be asked to sign a data-sharing agreement, subject to UCSF policies.
